# From suicidal ideation to hope: Perceived mental health transformation following a culturally adapted multiple family group therapy for adolescents living with HIV in Ghana - A qualitative process evaluation

**DOI:** 10.1371/journal.pmen.0000600

**Published:** 2026-11-10

**Authors:** Dorothy Serwaa Boakye, David Atsu Deegbe, Gladys Dzansi, Adote Anum, Samuel Adjorlolo

**Affiliations:** 1 Department of Health Administration and Education, University of Education, Winneba, Ghana; 2 Department of Mental Health Nursing, School of Nursing and Midwifery, University of Ghana, Accra, Ghana; 3 Department of Adult Health Nursing, School of Nursing and Midwifery, University of Ghana, Accra, Ghana; 4 Department of Psychology, School of Social Sciences, University of Ghana, Accra, Ghana; 5 Research and Grant Institute of Ghana, Legon, Ghana; PLOS: Public Library of Science, UNITED KINGDOM OF GREAT BRITAIN AND NORTHERN IRELAND

## Abstract

Adolescents living with HIV (ALHIV) in sub-Saharan Africa experience profound mental health challenges including depression, anxiety, and suicidal ideation. Evidence-based psychological interventions specifically designed for this population remain scarce, particularly family-based approaches in resource-limited settingsThis study explored ALHIV's perceived mental health transformation following participation in culturally adapted Multiple Family Group Therapy (MFGT) in Ghana.Qualitative study nested within a randomized controlled trial (NCT06701942) conducted in Lower Manya Krobo District, Ghana. Nineteen ALHIV who completed the 10-MFGT core content sessions. MFGT intervention participated in three focus group discussions. Data were analyzed using Braun and Clarke's reflexive thematic analysis.Three major themes emerged: (1) Pre-Intervention Mental Health Crisis, (2) Psychological Change Mechanisms, and (3) Mental Health Transformation. Pre-intervention, ALHIV described experiencing profound mental health crisis-level distress including active suicidal ideation with method-specific planning, emotional dysregulation, and existential foreclosure. Intervention mechanisms facilitating transformation included cognitive reframing through culturally resonant metaphors, dual-level universality (peer and intergenerational family), biomedical knowledge (U = U) as psychological intervention, and emotional catharsis in safe spaces. Post-intervention, participants described substantial perceived improvements in mental health characterized by reduced suicidal ideation, emotional regulation gains, reduced anxiety, and greater psychological peace.Culturally adapted family-based group therapy was associated with meaningful mental health improvements, including reduced suicidal ideation. Participants described shifts in emotional wellbeing, coping, and family cohesion, though the qualitative design precludes causal claims. Findings support the feasibility of task-shifted, family-centered interventions in resource-limited settings and underscore the need to integrate mental health screening and family-based support into HIV diagnosis protocols.

Trial registration

Clinical trials.gov (NCT06701942)

## Introduction

Adolescents living with HIV (ALHIV) face a disproportionate mental health burden compared to HIV-negative peers, with depression prevalence ranging from 20–50% globally, creating a syndemic relationship wherein mental health problems and HIV compound negative outcomes [1–4]. Depression among ALHIV is consistently associated with poor antiretroviral therapy (ART) adherence, disease progression, higher viral loads, and reduced quality of life [5–7]. Beyond depression, ALHIV experience elevated rates of anxiety, post-traumatic stress disorder, behavioral problems, and substance use [8–10]. Particularly alarming is suicidal ideation prevalence ranging from 15–35% in sub-Saharan Africa [11–13]. In Ghana, significant psychological distress among ALHIV, including depression, anxiety, and suicidal thoughts, is often precipitated by HIV diagnosis disclosure and exacerbated by HIV-related stigma [14–16].

Unique contextual vulnerabilities intensify this burden. Pervasive stigma creates social rejection and isolation that profoundly damage psychological wellbeing [17,18], while many ALHIV face status disclosure during adolescence, a period of heightened psychological vulnerability [19]. Limited family communication about HIV, caregiver mental health problems, poverty, educational disruption, and orphanhood create cumulative risk factors producing severe mental health crises [20–22]. Notably, family conflict and poor caregiver-adolescent communication have been directly linked to elevated suicidal ideation among ALHIV, positioning family functioning as both a risk factor for, and a lever against, adolescent suicidality [21–23]. Without intervention, these challenges persist into young adulthood, compromising HIV treatment outcomes, sexual and reproductive health, and life trajectories [24,25].

Despite well-documented need, evidence-based psychological interventions specifically designed for ALHIV remain scarce in low- and middle-income countries [26,27]. A recent systematic review confirms that few mental health interventions for ALHIV in low- and middle-income countries have been rigorously evaluated, and even fewer incorporate trauma-informed principles despite the high burden of cumulative trauma in this population [28,29]. Most HIV care programs in sub-Saharan Africa focus predominantly on biomedical management, with mental health needs either unrecognized or addressed through ad hoc counseling lacking theoretical grounding or empirical validation [30,31]. Interventions developed in high-income settings face significant challenges to adaptation: individual psychotherapy models require professional mental health workforce capacity that is lacking in most sub-Saharan African countries [32]. Ghana has fewer than two psychiatrists per million population, with limited availability of psychologists trained in evidence-based psychotherapies [33]. Western-developed interventions also inadequately address the cultural, familial, and spiritual dimensions of mental health valued in Ghanaian contexts [34]. These workforce shortages and infrastructural gaps constitute the core implementation constraints that any scalable mental health intervention for ALHIV in Ghana must address.

Family-based interventions represent a promising approach for ALHIV mental health. The family is the primary context for adolescent development and the main source of social support in African cultures, making family-inclusive interventions particularly well suited to this setting [35,36]. Caregiver mental health, family communication quality, and caregiver-adolescent relationship dynamics significantly influence ALHIV psychological outcomes [23,37]. Several family-based and multiple-family-group models have been developed and evaluated in sub-Saharan Africa, including family-strengthening and economic-support interventions for HIV-affected families in Uganda [36,38] and the VUKA family program in South Africa [39], and broader reviews confirm the value of family-inclusive approaches for ALHIV [29]. This study addresses a narrower, unaddressed gap: culturally adapted Multiple Family Group Therapy targeting suicidal ideation and severe mental health crisis among ALHIV has not been evaluated through in-depth qualitative process evaluation in a Ghanaian context, and existing African family interventions have not documented participants’ lived experience of psychological transformation in sufficient depth to inform cultural adaptation and scale-up.

Given the workforce shortages and infrastructural gaps described above, Multiple Family Group Therapy (MFGT) addresses both ALHIV mental health needs and these implementation constraints of resource-limited settings by task-shifting delivery to trained lay health workers and peer caregivers rather than scarce specialist providers. By bringing together multiple families affected by a common challenge in structured group sessions, MFGT combines the therapeutic mechanisms of family therapy, improving communication, restructuring relationships, and addressing family dynamics, with those of group therapy, including peer support, universality, vicarious learning, and hope instillation [40–42]. Grounded broadly in family systems theory, which conceptualizes psychological problems as embedded within and maintained by family interaction patterns rather than residing solely within individuals, and drawing specifically on Minuchin's Structural Family Therapy for its emphasis on family structure, roles, hierarchy, and communication patterns [43], MFGT directly addresses family communication breakdowns, enhances mutual understanding, and strengthens family as a protective factor for adolescent mental health [44]. This structural lens informed the present study's attention to family roles, communication patterns, and relational boundaries as both contributors to, and pathways of recovery from, adolescent mental health crisis. Simultaneously, the multi-family format provides ALHIV with peer connections that challenge existential isolation [45]. MFGT and related family-based group models have demonstrated effectiveness for adolescent behavioral health challenges in high-income countries, including depression, substance use, and chronic illness management [46–48], and, increasingly, in sub-Saharan African settings, including family-strengthening interventions in Uganda and the VUKA family program in South Africa [35,36,39]. Applying this specific culturally adapted model to ALHIV in the Ghanaian context required cultural adaptation to align with local values, belief systems, family structures, and resource realities [49,50].

Despite MFGT's promise, qualitative evidence documenting participants’ lived experiences of mental health transformation remains limited. In this study, mental health transformation refers to participant-narrated shifts in psychological state and subjective wellbeing, including changes in suicidal ideation, emotional regulation, anxiety, and hope, as expressed in participants’ own accounts, rather than clinically diagnosed or biomarker-verified change. Understanding how ALHIV and their families experience and make meaning of psychological change is essential for interpreting intervention mechanisms, identifying active therapeutic ingredients, and illuminating pathways from distress to recovery that quantitative measures may not fully capture, particularly for interventions addressing severe outcomes like suicidal ideation.

This study therefore explores mental health transformation experienced by ALHIV following participation in a culturally adapted MFGT in Ghana's Lower Manya Krobo District. Through thematic analysis of post-intervention focus group discussions, we address: (1) What mental health challenges did ALHIV experience before MFGT? (2) What intervention mechanisms did participants identify as facilitating mental health change? (3) What mental health transformations did participants experience following the intervention? By centering ALHIV voices, this study contributes essential qualitative evidence on processes and outcomes of family-based mental health intervention in African contexts, with implications for intervention refinement, implementation, and scale-up.

## Methods

### Ethics consideration

Ethical approval for this study was obtained from the Ghana Health Service Ethics Review Committee (GHS-ERC 004/07/24) prior to participant recruitment and data collection. The study adhered to all ethical guidelines for research involving minors, including obtaining written informed consent from caregivers and written assent from adolescents. Additional safeguards were implemented given the sensitive nature of mental health discussions and the vulnerable status of ALHIV, including provisions for referring participants experiencing acute psychological distress to available counseling services.

### Study design

This qualitative study was nested within the parent Abusuafoɔ Apomuden (“Healthy Families”) trial (NCT06701942) evaluating a culturally adapted Multiple Family Group Therapy (MFGT) intervention for ALHIV in Ghana. The parent trial employed a two-arm, cluster-randomised controlled trial design, in which the health facility (clinic) served as the unit of randomisation, to evaluate the effects of this programme on depressive symptoms, anxiety symptoms, and antiretroviral therapy (ART) adherence among young people living with HIV (YPLHIV) and their caregivers. Atua Government Hospital was allocated to the intervention arm and Asesewa Government Health Facility to the control arm using a computer-generated randomisation sequence [51], with allocation concealment maintained by withholding clinic allocation from research team members conducting baseline assessments until enrolment was complete at both sites; randomisation and allocation were finalised by 31 May 2025. As only two clusters (one per arm) were randomised, the design provides no within-arm cluster replication. Primary outcomes (depression, anxiety, and ART adherence) were measured using standardised psychometric instruments (PHQ-9, GAD-7, and a validated adherence measure) at baseline and follow-up. The trial's development and evaluation drew on complementary theoretical frameworks applied across different phases: the Theory of Triadic Influence, Social Action Theory, and Stirman's adaptation framework guided the cultural adaptation of the intervention; family systems theory guided its pilot implementation; and the RE-AIM framework guided the evaluation phase. The trial was prospectively registered on ClinicalTrials.gov (NCT06701942) on 22 November 2024, prior to participant enrolment commencing on 10 December 2024. At the time of writing, the parent trial has completed data collection and is in the analysis phase; quantitative outcome findings will be reported separately. The reporting of the qualitative component of the trial follows the Consolidated Criteria for Reporting Qualitative Research (COREQ) [52] (S1 Checklist) and the reporting of the trial follows the CONSORT 2025 checklist items applicable to this study [53] (S2 Checklist)

### Study setting

The study was conducted in Lower Manya Krobo District of Ghana's Eastern Region, a predominantly rural area with high HIV prevalence [54]. The district has experienced generalized HIV epidemics since the 1980s, with significant numbers of perinatally infected children now reaching adolescence [55]. Healthcare services are provided through Atua Government Hospital, Asesewa Government Health Facility, and other health centers offering antiretroviral therapy, though mental health services are minimal with no dedicated psychiatric services.

### The “Abusuafoɔ Apomuden” intervention

In line with the TIDieR (Template for Intervention Description and Replication) checklist [56], the following description addresses the intervention's rationale, materials, procedures, providers, mode and setting of delivery, dose, tailoring, and fidelity. “Abusuafoɔ Apomuden” (“Healthy Families”) is a systematic cultural adaptation of the “4Rs and 2Ss” Multiple Family Group Therapy model originally developed by McKay and colleagues [40], organised around the core components of Rules, Responsibility, Relationships, and Respectful communication (the “4Rs”) and Stress and Social support (the “2Ss”), and designed to address family communication, HIV-related stigma, and adolescent mental health through combined family-systems and group-therapy mechanisms. The intervention was adapted directly for the Ghanaian context by the study team rather than through an intermediate regional adaptation. The adaptation was guided by the Theory of Triadic Influence [57], Social Action Theory [58], and the Stirman et al. adaptation coding framework [59], and proceeded through a structured, four-phase community stakeholder engagement process completed between September 2024 and February 2025. This process involved mapping and recruitment of 34 community stakeholders, comprising caregivers, older family members, adolescents and young adults, family heads, healthcare professionals, and Community Health Officers; iterative review and revision of the intervention curriculum to reflect locally resonant metaphors, language (Twi and Dangbe), and therapeutic activities; and final stakeholder endorsement of the adapted manual. The full adaptation process is described in a separate methodological paper [60].

Intervention materials comprised structured facilitator manuals, session guides, locally adapted psychoeducational materials, and take-home assignments. Participants at the intervention site received the adapted MFGT intervention face-to-face and in-person, in addition to usual HIV care. The intervention comprised 11 structured group sessions of approximately 120 minutes each, delivered weekly on Sundays at Atua Government Hospital, in groups of 10–12 families (4 cohorts), group sizes consistent with those used in the South African VUKA [39] and Ugandan Amaka Amasanyufu [61] adaptations of the 4Rs and 2Ss framework. Each enrolled adolescent attended every session together with their participating caregiver; sessions were not split into separate adolescent-only or caregiver-only components, and family-level activities were incorporated into every session rather than confined to a subset of sessions. The first session served as an orientation and community-building meeting, in which group rules and the meeting schedule were established collectively. The subsequent ten sessions addressed six therapeutic domains mapped onto the 4Rs and 2Ss structure: respectful communication within and between families; building and mobilising social support networks; stress identification and management; family rules, responsibilities, and collective accountability; family relationships, trust-building, and conflict resolution; and HIV stigma psychoeducation and cognitive reframing, culminating in an eleventh session comprising a graduation ceremony with certificate presentation and sustainability planning. Sessions used psychoeducation, structured group discussion, family communication skills training, role-play, drama, storytelling, and family-based problem-solving exercises, alongside drumming, singing, prayer, local proverbs, music, and dance, delivered bilingually in Twi and Dangbe as required by participants. Session content was tailored to family-specific needs and cultural dynamics within a fixed manualised structure; two modules (Rules, and Respectful Communication) received additional reinforcement across cohorts in response to attendance and family-conflict patterns observed during delivery. Data collection for this qualitative component began after the tenth session, once core intervention content had been delivered.

Sessions were co-facilitated by trained parent peers, caregivers with lived experience of HIV caregiving in the local community, and health workers holding a minimum diploma or bachelor's degree in a relevant field, at least two years of experience working with HIV-affected families, and fluency in English and Dangbe. All facilitators completed a three-day intensive training workshop covering the theoretical and evidence base of the programme, facilitation skills, cultural competency specific to the Krobo context, and ethical considerations. Fidelity was supported through structured session manuals with scripted activities, retaining flexibility to respond to emergent family needs, alongside ongoing weekly group supervision, audio recording of sessions for supervisory review, random unannounced session observation, weekly attendance reviews, and monthly facilitator logs; formal fidelity checklists were not the focus of this qualitative component and are reported separately as part of the parent trial's process evaluation.

### Participants and recruitment

Recruitment and enrollment of ALHIV and their caregivers into the parent two-arm cluster-randomised controlled trial commenced on 10^th^ December 2024 and were completed on 31^st^ May 2025. The parent trial randomized clusters to either the MFGT intervention arm or a standard-of-care control arm; participants in the present qualitative component were drawn exclusively from the intervention arm, as the focus group discussions were designed specifically to explore experiences of MFGT participation. This qualitative process evaluation was scoped and funded specifically to explore ALHIV's and caregivers’ experiences of MFGT participation and to surface participant-perceived mechanisms of change; it was not designed to establish comparative effectiveness against the control arm, which is being assessed through the parent trial's quantitative outcome analysis. Participants were ALHIV who had completed at least 7 of the 10 MFGT core content sessions, the threshold used to define adequate intervention exposure. Nineteen adolescents participated across three focus group discussions (n = 6, 6, 7). Eligibility criteria included: (1) age 10–22 years, (consistent with the WHO/UNAIDS extended definition of adolescents and young people, which encompasses ages 10–24 for HIV programming and research purposes [62]), (2) confirmed HIV-positive status, (3) receiving ART services at Atua Government Hospital, (4) living with a caregiver willing to participate, and (5) able to provide assent/consent. Of the intervention-arm ALHIV meeting this attendance threshold, purposive sampling ensured diversity in age, gender, and cohort assignment (i.e., which of the four MFGT cohorts the participant belonged to). Facilitators invited eligible adolescents during the final intervention session. Participation was voluntary, with assurances that declining would not affect HIV care.

### Data saturation

Data saturation was assessed iteratively using a codebook developed inductively from the first transcript and applied consistently across subsequent transcripts by the same two coders (DSB and SA), with saturation monitored through structured team debriefs held after each FGD to compare newly generated codes against the existing codebook. After two focus groups (n = 12), preliminary analysis identified emerging themes, and a third group (n = 7) confirmed saturation. The third FGD corroborated themes from the first two groups, with participants articulating similar pre-intervention mental health challenges, intervention mechanisms, and post-intervention transformations. While individual stories varied, core transformation patterns were consistently represented, with no substantively new themes emerging beyond minor variation in illustrative detail. Thematic saturation was achieved with 19 participants across three groups, consistent with recommendations for qualitative research in homogeneous populations [63]. Given the breadth of topics covered (suicidality, family relationships, stigma, hope, and adherence), saturation is interpreted here as domain-specific (achieved for the recurring patterns reported) rather than as an exhaustive account of every possible theme within this population. (Fig 1).

**Fig 1:**
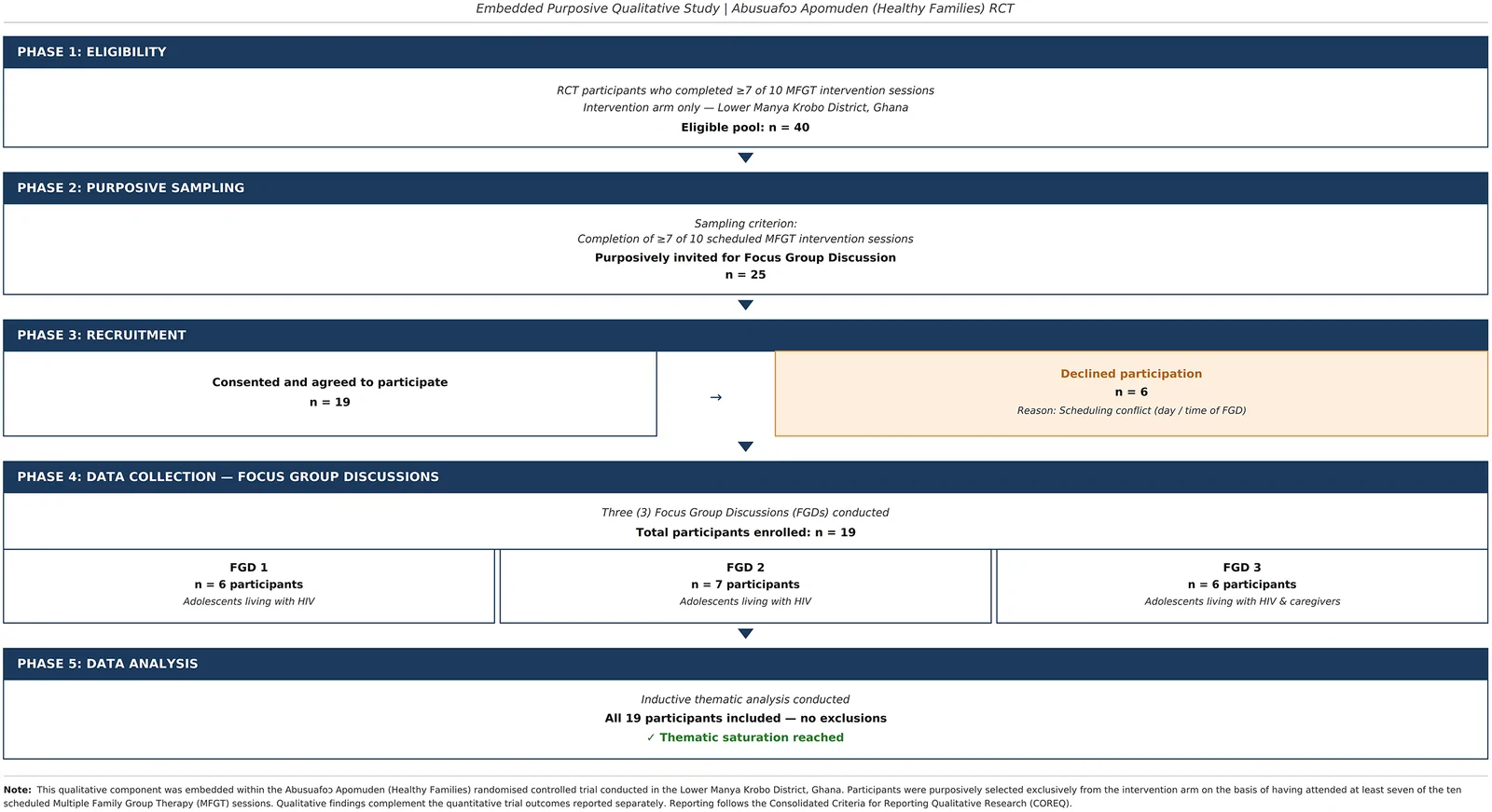
Qualitative component flow diagram.

### Data collection

Focus group discussions (FGDs) were conducted face to face following completion of the 10-core content sessions of the MFGT intervention (Immediate post intervention). FGD sessions were led by the 1st author with crucial support from a research assistant who performed dual functions: providing immediate language interpretation between English and local languages (primarily Dangme and Twi) as needed, while simultaneously documenting session notes. This bilingual support ensured all participants could fully engage regardless of their language preference. The facilitator was not involved in intervention delivery to reduce social desirability bias and encourage candid feedback.

Each FGD lasted approximately 60–90 minutes and was conducted in a private conference room at Atua Government Hospital (the intervention site) to ensure confidentiality and minimize distractions.

A semi-structured FGD guide (S1 Text) was developed based on the study's research questions and MFGT intervention components. The guide included open-ended questions exploring: (1) mental health experiences before the intervention; (2) experiences during the MFGT sessions; (3) perceived changes in mental health, family relationships, and treatment adherence. Probing questions were used to elicit deeper reflection and specific examples.

All FGDs were audio-recorded with participants’ permission. Before recording, facilitators reiterated confidentiality expectations, explained how data would be used, and obtained written assent/consent. Participants were assured they could decline to answer any question or stop participation at any time. At the conclusion of each FGD, participants received refreshments and transportation reimbursement as tokens of appreciation for their time.

### Data analysis

Audio recordings were transcribed verbatim in the language spoken (Twi, Dangbe, English) then translated into English. Back-translation of sample transcripts verified translation accuracy. Transcripts were de-identified with participant names replaced by codes and identifying details removed. Data were analyzed using Braun and Clarke's six-phase reflexive thematic analysis [64], emphasizing researcher interpretation and meaning-making. Two authors (DSB and SA) familiarized themselves with data by reading transcripts multiple times while listening to audio recordings to capture tone and emotion, noting initial observations in a research journal. Transcripts were imported into QDA Miner (Provalis Research, Montreal, QC, Canada) [65] for systematic coding, with descriptive codes applied inductively to data segments including pre-intervention mental health states (suicidal ideation, crying, fear), intervention mechanisms (learning U = U, peer support, family communication), and post-intervention outcomes (hope, peace of mind, anxiety reduction).

Codes were examined for patterns, with related codes grouped into potential themes and sub-themes. For example, codes for suicidal thoughts, crying episodes, fear, and worry were grouped under “Pre-intervention Psychological Distress.” Potential themes were reviewed against coded data and entire transcripts to ensure accurate representation and coherent patterns, with some themes collapsed, split, or discarded following research team discussions. Final themes were refined and clearly defined to capture distinct aspects of mental health transformation, with sub-themes capturing nuance. Themes were organized into a coherent narrative tracing the transformation trajectory from pre-intervention distress through intervention mechanisms to post-intervention outcomes, with illustrative quotes selected that were vivid, clear, and representative.

### Rigor and trustworthiness

Multiple strategies aligned with Lincoln and Guba's criteria ensured quality [66]. Credibility was enhanced through prolonged engagement with data (multiple transcript reviews), peer debriefing sessions where the research team discussed emerging themes, and member checking with a subset of participants to verify resonance. Transferability was addressed through thick description of study context, participant characteristics, intervention details, and analytical processes. Dependability was established through detailed audit trails documenting analytical decisions, theme development, and interpretive shifts. Confirmability was pursued by grounding themes in participant quotes, selecting diverse representative examples, and using QDA Miner software to systematically track coding decisions.

### Reflexivity and researcher positionality

Reflexivity, critical examination of how researchers’ backgrounds shape data collection and interpretation, was central to analytical integrity [67]. The lead researcher (Dorothy) is a Ghanaian PhD candidate in implementation science with extensive HIV research experience and deep familiarity with Lower Manya Krobo District. This insider status provided cultural competence for interpreting narratives and recognizing culturally specific expressions of distress, but required vigilance against over-familiarity. Dorothy's commitment to improving ALHIV mental health services created investment in demonstrating intervention effectiveness, potentially biasing interpretation toward positive outcomes. To counter this, she maintained a reflexive journal documenting assumptions and emotional responses, with regular team discussions from diverse disciplinary backgrounds (Adolescent mental health, clinical psychology, family therapy and HIV care) providing external perspectives challenging interpretation. The team's affiliation with the intervention site potentially introduced social desirability bias. To mitigate this, FGDs were facilitated by researchers not involved in intervention delivery, confidentiality was emphasized, and participants were encouraged to share both positive and negative experiences. The team embraced positioned subjectivity while implementing systematic safeguards against interpretive bias.

## Findings

### Sociodemographic characteristics of participants

Table 1 summarizes the sociodemographic characteristics of the 19 adolescent participants. The majority of the participants were aged 16–22 years, predominantly female (73.7%), and most were students (63.2%). Educational attainment varied, with the largest proportion currently in junior high school. Primary caregivers were mainly mothers and siblings. Nearly half had been diagnosed with HIV for 12–15 years, reflecting predominantly perinatal acquisition of HIV among these longer-diagnosed participants, while other participants acquired HIV later through sexual transmission, and most had not previously participated in support programs.

**Table 1:** Sociodemographic Characteristics of Adolescent Participants (N = 19).

Variable	Range/Categories	Frequency (n)	Percentage (%)
Age (years)	10–15	7	36.8
	16-22	12	63.2
Gender	Female	14	73.7
	Male	5	26.3
Education Level	Primary	3	15.8
	JHS (in progress)	6	31.6
	JHS (completed)	3	15.8
	SHS (in progress)	4	21.1
	SHS (completed)	3	15.8
Current Occupation	CAT (Adolescent Treatment Supporter)	2	10.5
	Unemployed	1	5.3
	Sewing/seamstress	2	10.5
	Vendor	2	10.5
	Student	12	63.2
Primary Caregiver	Mother	6	31.6
	Grandmother	3	15.8
	Aunt	3	15.8
	Grandfather	1	5.3
	Brother	1	5.3
	Sister	5	26.3
Time Since HIV Diagnosis	<1 year	4	21.1
	1–2 years	6	31.6
	12–15 years	9	47.4
Prior Support Programs	Yes	2	10.5
	No	17	89.5

## Main findings

### Themes and subthemes

Three overarching themes and twelve subthemes emerged from the thematic analysis, tracing participants’ transformation trajectory from pre-intervention psychological crisis through intervention mechanisms to post-intervention mental health outcomes (Table 2). Fig 2 summarizes the psychological change mechanisms as perceived and reported by participants; it depicts a descriptive summary of participant accounts rather than an empirically established causal pathway, given the absence of a comparison group.

**Table 2:** Themes, subthemes, and illustrative quotes from thematic analysis.

Theme	Subtheme	Description	Illustrative Quote
**Theme 1: Pre-Intervention Mental Health Crisis**	**1.1 Suicidal Ideation: From Passive Death Wishes to Active Planning**	Participants described a spectrum of suicidal thoughts following HIV diagnosis, from passive death wishes to active method-specific planning, including environmental assessment for feasibility.	“When I was first diagnosed, I thought of attempting suicide but our ceiling at home is concrete so, I couldn't hang myself. But I kept thinking of other ways to do it...” **(P3)**
	**1.2 Emotional Distress Symptoms**	Frequent uncontrollable crying episodes, hypervigilance about HIV-related conversations, paranoid ideation about accidental disclosure, and pervasive anxiety about social judgment.	“For the first time I was told of my diagnosis I really wept and also at times when people talk about the sickness, I feel they are talking about me because they know my status...” **(P2)**
	**1.3 Mental Health Risk Factors**	Convergence of multiple risk factors: HIV diagnosis-triggered suicidal ideation, parental verbal abuse reinforcing HIV as shameful, classroom HIV education triggering anxiety, and familial isolation.	“I used to cry a lot because my dad insults me that I have HIV.” **(P3)**
**Theme 2: Psychological Change Mechanisms**	**2.1 Cognitive Reframing and Normalization**	MFGT facilitators taught adolescents to reconceptualize HIV using culturally resonant normalizing comparisons (e.g., malaria, diabetes), dismantling catastrophic thinking through collective meaning-making.	“I have come to accept that we are normal people, there's no difference between us and those who do not have the virus.” **(P5)**
	**2.2 Universality and Peer Support**	The multi-family group format provided dual-level universality: horizontal (peer-to-peer) and vertical (intergenerational family), challenging existential isolation and normalizing treatment adherence.	“I used to think that I was the only one who takes the drugs... ever since I found out that my mom and my aunties also take the drug, I am very okay.” **(P11)**
	**2.3 Hope Restoration Through Biomedical Knowledge (U = U)**	Education on Undetectable = Untransmittable (U = U) and prevention of mother-to-child transmission directly reversed existential foreclosure, restoring aspirations for marriage and parenthood.	“Initially I had made up my mind that I was not going to get married, but after the lesson last week I learned that if you take your drugs well, you can get married...” **(P3)**
	**2.4 Psychoeducation on Mental Health**	Explicit teaching on the stress-suicide link and rumination as a modifiable risk factor equipped adolescents with practical strategies (e.g., thought diversion) and a sense of agency over psychological experiences.	“Sir Randy taught us not to overthink things because it can cause us to do terrible things like attempting suicide, and so if something worries us we shouldn't overthink it...” **(P3)**
	**2.5 Emotional Catharsis in a Safe Space**	Culturally resonant therapeutic activities (drumming, singing, dancing, prayer) within a confidential group setting facilitated non-verbal cathartic release of accumulated grief, stress, and trauma.	“There are days my mom does things that get me angry at home and so, when I come here for the program, the therapeutic activities such as drumming, singing and dancing makes me feel relieved.” **(P5)**
**Theme 3: Post-Intervention Mental Health Transformation**	**3.1 Suicidal Ideation Cessation**	Complete resolution of suicidal thoughts, with HIV reconceptualized as a manageable chronic condition. Participants reported freedom from HIV-related preoccupation and restoration of psychological peace.	“Our coming here has helped me to change my mind and stop thinking about suicide and have taken this as a regular sickness like malaria.” **(P3)**
	**3.2 Emotional Regulation Achievement**	Universal cessation of frequent crying episodes and HIV-triggered emotional dysregulation, indicating successful grief resolution and development of effective coping strategies.	“For the first time I was told of my diagnosis I really wept... but based on what I have learnt so far, I don't mind anymore.” **(P2)**
	**3.3 Anxiety and Fear Elimination**	Meaningful reduction in HIV-related anxiety enabled participants to reclaim previously abandoned normative adolescent activities, demonstrating behavioral change and social reintegration alongside internal psychological shifts.	“Initially, I do not go for dance practice anytime we were called because I used to think people would gossip about me, but now I go for dance practice, and I also perform.” **(P8)**
	**3.4 Psychological Peace and Well-being**	Beyond symptom reduction, participants described achieving positive psychological states — peace of mind, contentment, and future-oriented hope — indicating genuine flourishing rather than mere symptom management.	“I do not even have the time to be thinking about me having the virus or worrying about it. I am very okay, my mind is at peace now.” **(P17)**

P = Participant.

**Fig 2:**
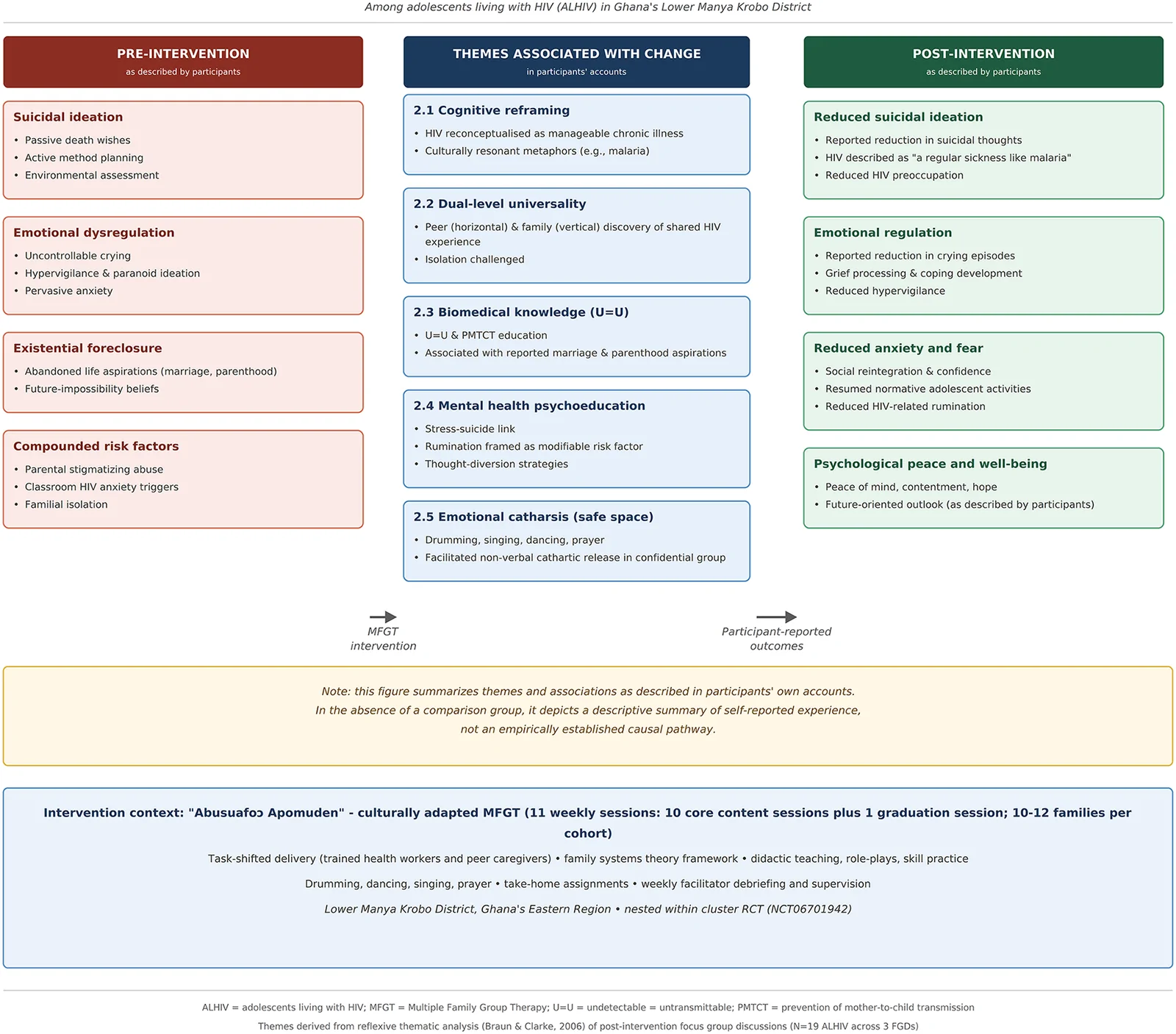
Participants reported themes in perceived psychological change following the culturally adapted multiple family group therapy.

### Theme 1: Pre-intervention mental health crisis

#### Subtheme 1.1: Suicidal ideation ranging from passive death wishes to active planning.

Participants described a spectrum of suicidal thoughts following HIV diagnosis, ranging from passive death wishes to active, method-specific suicide planning with environmental assessment and consideration of alternative methods when initial plans proved unfeasible.

“*Sometimes when I think about it [HIV diagnosis], I feel like dying because I ask myself why it should be me among all my peers. So sometimes I think it would be better if I just die*.” (P7)

“*When I was first diagnosed, I thought of attempting suicide but our ceiling at home is concrete so, I couldn't hang myself. But I kept thinking of other ways to do it since I'm not bold enough to use a knife. However, our coming here has helped me to change my mind and stop thinking about suicide and have taken this as a regular sickness like malaria which will definitely go*.” (P3)

These quotes reveal that HIV diagnosis triggered an existential crisis so profound that death seemed preferable to living with the condition. The participants’ suicidal ideation existed along a severity continuum: P7 expressed passive death wishes characterized by hopelessness and existential questioning (“why it should be me”), while P3 progressed to active suicide planning with method selection, environmental assessment, and persistent method-seeking when initial plans proved unfeasible. Both responses reflect a common underlying pattern: the internalization of HIV as an identity-destroying catastrophe rather than a manageable medical condition.

#### Subtheme 1.2: Emotional distress symptoms.

Adolescents experienced intense emotional suffering following HIV diagnosis, manifesting primarily as frequent and uncontrollable crying episodes. These emotional responses were accompanied by hypervigilance about HIV-related conversations, paranoid ideation regarding disclosure, and pervasive anxiety about social judgment and stigma.

“*For the first time I was told of my diagnosis I really wept and also at times when people talk about the sickness, I feel they are talking about me because they know my status, but based on what I have learnt so far, I don't mind anymore*.” (P2)

“*At first, I used to cry a lot but now I don't cry anymore*.” (P6)

These quotes reveal that HIV diagnosis precipitated intense grief reactions and pervasive anxiety that affected adolescents across all ages, including the youngest participant at 14 years old. The universality of crying episodes, described by both P2 and P6 as frequent and uncontrollable, suggests that emotional dysregulation was a core feature of the post-diagnosis experience, reflecting profound psychological distress rather than typical adolescent adjustment challenges.

#### Subtheme 1.3: Mental health risk factors.

Multiple risk factors converged to create conditions for severe mental health deterioration. HIV diagnosis directly precipitated suicidal ideation

“*When I was first diagnosed, I thought of attempting suicide*.” (P3)

Parental abuse amplified risk:

“*I used to cry a lot because my dad insults me that I have HIV*.” (P3)

Classroom HIV education triggered anxiety:

“*Anytime our teacher raises the topic of STI and HIV whilst we are learning in class, I feel like I am the one she's talking about but ever since we started coming for this training, I realized that I am not the one and neither has it been written on me that I have the virus*.” (P12)

Existential isolation within families intensified distress:

“*I use to get very angry and sad about just me having the virus in my family, because all my brothers do not carry the virus*.” (P15)

*“My grandma used to be very rude to me, she insults me at every giving opportunity and shout at me too. But then I realized that she has changed ever since we started this training, she doesn't shout at me or insult me indiscriminately anymore.*” (P16)

Multiple risk factors interacted to compound psychological distress among ALHIV. P3's experience illustrates this starkly: diagnosis-triggered suicidal ideation was exacerbated by ongoing parental verbal abuse that reinforced her HIV status as shameful. Rather than providing protective support, her father became an additional trauma source through continuous stigmatizing insults. This dual burden, carrying HIV and being actively shamed by a parent, likely intensified worthlessness and fueled progression to active suicide planning. P16's account of persistent verbal insults from a grandmother further illustrates that caregiver-perpetrated stigma was not isolated to a single family configuration, suggesting this risk factor operated across different caregiving arrangements. P12 and P15's accounts reveal how safe environments, classrooms and families, became psychological threats. P12's hypervigilance during HIV education demonstrates how well-intentioned public health messaging can paradoxically traumatize HIV-positive adolescents lacking cognitive frameworks to separate general education from personal exposure

### Theme 2: Psychological change mechanisms

#### Subtheme 2.1: Cognitive Reframing and normalization.

A fundamental mechanism of mental health change within the MFGT program was cognitive reframing of HIV from death sentence to manageable chronic illness. MFGT facilitators explicitly taught adolescents to reconceptualize HIV using normalizing comparisons (malaria, diabetes) and routine integration metaphors during psychoeducation sessions. This therapeutic reframing was reinforced through group discussions and peer testimonials throughout the 10-week core content of MFGT intervention.

Participants reframed HIV as a manageable chronic condition

“*However, our coming here has helped me to change my mind and stop thinking about suicide and have taken this as a regular sickness like malaria*” (P3)

“*I have come to accept that, we are normal people, there's no difference between us and those who do not have the virus*.” (P5)

These quotes demonstrate how the MFGT intervention systematically dismantled catastrophic thinking about HIV through cognitive restructuring and normalization strategies. P3's transformation from active suicide planning to viewing HIV as comparable to malaria represents a fundamental shift in illness perception: from terminal to treatable, from exceptional to ordinary. P5's assertion of normalcy (“there's no difference between us”) reflect successful internalization of the intervention's core message: HIV, while chronic, does not define one's identity or diminish one's humanity.

Collectively, these accounts reveal that psychoeducation combining accurate HIV information with normalizing metaphors successfully countered misinformation, reduced existential dread, and created psychological space for hope and future-orientation to emerge.

#### Subtheme 2.2: Universality and peer support.

The MFGT group format provided direct experience of not being alone with HIV. Throughout the 10-week program, seeing other adolescents living and thriving with HIV in the same group sessions challenged existential isolation and created mutual support networks that extended beyond the weekly meetings. This universality emerged as one of the most powerful therapeutic mechanisms of the MFGT intervention.

“*I was expecting to get encouragement from other adolescents, knowing that I am not the only one carrying the virus*…*Yes I did*” (P6)

“*I used to think that I was the only one who takes the drugs and so that makes me scared but ever since I found out that my mom and my aunties also take the drug, I am very okay. I feel confident taking the drugs now and so everything is okay with me mentally*.” (P11)

These quotes reveal how the discovery of universality, realizing others share one's HIV status, served as a powerful antidote to the existential isolation that characterized participants’ pre-intervention experience. The MFGT group format uniquely provided what individual counseling could not: tangible, embodied evidence that other adolescents and family members were living with HIV, thereby challenging participants’ beliefs about being uniquely afflicted.

The realization of shared experience operated at multiple levels. Within the peer group, and within families. This dual discovery, of peer and family universality, reduced stigma, normalized treatment adherence, and provided comprehensive psychological benefits. Universality thus functioned as both a cognitive intervention (challenging catastrophic beliefs about singular affliction) and an emotional one (providing belonging and mutual support), making it central to the MFGT's therapeutic effectiveness in restoring mental health and hope.

#### Subtheme 2.3: Hope Restoration through knowledge.

MFGT psychoeducation sessions on Undetectable = Untransmittable (U = U), normal life expectancy with treatment adherence, and prevention of mother-to-child transmission directly reversed existential foreclosure and restored hope for the future. These biomedical education components were delivered by MFGT facilitators during dedicated psychoeducation sessions within the 10-week program.

“*Initially I had made up my mind that I was not going to get married, but after the lesson last week I learned that if you take your drugs well, you can get married, and even if you have sex with your wife, she is not going to contract the virus*.” (P5).

*“I had also told myself growing up that I was not going to give birth because I thought my child was going to be infected, but because of the training program, I now know that once your virus becomes undetectable, you can give birth and your child will not get HIV.*” (P4)

These quotes demonstrate how accurate biomedical information about HIV transmission prevention directly dismantled catastrophic beliefs about future impossibility and restored hope for normative life milestones. Both participants had pre-intervention foreclosed on fundamental life aspirations, marriage and parenthood, based on the assumption that HIV inevitably meant transmitting the virus to partners and children. P5 focused specifically on marriage and sexual intimacy, while P4 addressed the parallel fear of transmitting HIV to a future child; together they illustrate that existential foreclosure extended across multiple normative life domains rather than being confined to a single aspiration. Learning about U = U and prevention of mother-to-child transmission through adherence transformed their future orientation from resigned hopelessness to active possibility. This restoration of future possibilities represents a fundamental shift from existential hopelessness to agency and self-determination.

#### Subtheme 2.4: Psychoeducation on mental health.

Explicit teaching about the stress-suicide link, rumination as risk factor, and mental health service availability was delivered during the session on stress management. The MFGT facilitator handling this module provided frameworks for understanding psychological experiences and strategies for managing distress. Building on the mental health literacy foundation established in week 2, these sessions ensured adolescents were well-equipped to cope with stress.

“*Sir Randy taught us not to overthink things because it can cause us to do terrible things like attempting suicide, and so if something worries us we shouldn't overthink it but we should find ways* (such as watching favorite TV shows*) of diverting our thoughts*” (P3)

*“We have been taught here that anytime you have a problem or you are depressed you need counseling or therapy and so that has helped me to manage my mental health.*” (P16)

These quotes reveal how mental health psychoeducation provided adolescents with both understanding of psychological risk mechanisms and practical tools for distress management. Participants learned to recognize rumination (“overthinking”) as a modifiable risk factor for suicidal behavior rather than an uncontrollable mental process, and were equipped with concrete strategies such as thought diversion to interrupt dangerous cognitive patterns. Beyond self-management strategies, P16's account indicates that psychoeducation also normalized formal help-seeking, framing counseling and therapy as legitimate responses to psychological difficulty rather than as sources of additional stigma. This reframing of mental processes as manageable rather than inevitable gave adolescents a sense of agency over their psychological experiences

#### Subtheme 2.5: Emotional Catharsis in safe space.

The MFGT intervention provided rare opportunity for emotional expression in a safe, confidential environment specifically designed for HIV-affected families. Therapeutic activities integrated into MFGT sessions, including singing, dancing, and prayer, facilitated cathartic release, while structured group sharing exercises allowed processing of grief and trauma. The enclosed meeting space and facilitator warmth created psychological safety throughout the 10-week program

“*There are days my mom does things that get me angry at home and so, when I come here for the program, the therapeutic activities such as drumming, singing and dancing makes me feel relieved*.” (P5)

*“Praying. Anytime I am not happy and I pray, I feel relieved. I become joyful.*” (P8)

*“I sing at church and so anytime they do something at home which gets me sad or when I am stressed, I just go inside and sing to cheer myself up.*” (P19)

These quotes reveal how the MFGT intervention created a therapeutic sanctuary where adolescents could safely process emotions that had no outlet in their daily lives. Catharsis was achieved through multiple modalities: P5 described relief through drumming, singing, and dancing; P8 through prayer; and P19 through singing as a self-initiated coping strategy she continued to use at home between sessions. The integration of culturally resonant activities provided non-verbal channels for emotional release, allowing participants to discharge accumulated stress and anger through embodied expression rather than cognitive processing alone. This cathartic function was particularly valuable for adolescents who may have lacked vocabulary or permission to articulate their emotional distress verbally

### Theme 3: Mental health transformation

#### Subtheme 3.1: Suicidal ideation cessation.

Post-intervention, participants described fundamental cognitive shifts characterized by reduced HIV-related fear, reduced rumination, and greater mental peace in their own accounts. These self-reported changes extended beyond acute crisis resolution to what participants described as sustained psychological wellbeing, with HIV appearing to no longer dominate participants’ mental and emotional landscapes, as narrated in these post-intervention accounts. Participants described the intervention as having transformed HIV from an unbearable existential threat into a manageable condition integrated into daily life.

A Participant described complete resolution of suicidal thoughts

“*When I was first diagnosed, I thought of attempting suicide but our ceiling at home is concrete so, I couldn't hang myself. But I kept thinking of other ways to do it since I'm not bold enough to use a knife. However, our coming here has helped me to change my mind and stop thinking about suicide and have taken this as a regular sickness like malaria*.” (P3)

HIV-related preoccupation was replaced by mental tranquility:

“*I do not even have the time to be thinking about me having the virus or worrying about it. I am very okay my mind is at peace now*.” (P15)

P3's and P15's accounts illustrate a striking shift from active suicidal crisis to a self-described state of psychological peace, among the most striking mental health accounts in this study. P3 described the intervention as having reshaped her illness perception, reducing the cognitive foundation that had earlier made death seem preferable to living with HIV. P15's description of mental peace and reduced HIV-related preoccupation suggests, in her own words, an experience extending beyond symptom reduction toward a broader sense of psychological relief. These self-reported shifts are consistent with, though cannot on their own establish, sustained clinical improvement or suicide prevention in a formal sense; without longitudinal follow-up or a comparison group, we can report participants’ own accounts of change but not verify their durability or attribute them definitively to MFGT rather than other concurrent factors.

#### Subtheme 3.2: Emotional regulation achievement.

Adolescents who reported pre-intervention crying described reduced or stopped crying following the MFGT program, in their own accounts, consistent with the intervention having contributed to grief processing and emotional coping development, though this was not independently verified.

“*For the first time I was told of my diagnosis I really wept and also at times when people talk about the sickness, I feel they are talking about me because they know my status, but based on what I have learnt so far, I don't mind anymore*.” (P2)

“*When it comes to the issue of worrying, I do not do that anymore, and the crying over me having the virus too have stopped*.” (P12)

These quotes illustrate two distinct routes to emotional regulation that participants associated with the intervention. P2's shift from acute grief and paranoid hypervigilance (“I feel they are talking about me”) to psychological indifference (“I don't mind anymore”) suggests emotional processing and cognitive reframing that reduced both her emotional reactivity and distorted thinking. P12's account extends this pattern: having earlier described believing classroom HIV education was directed at her personally, she went on to report that both her worrying and crying had stopped, suggesting her reduced hypervigilance was accompanied by broader emotional relief. Together, these accounts suggest reduced crying was associated, in participants’ own attributions, with the intervention addressing underlying grief and building coping mechanisms, though this pattern was not systematically assessed across all participants.

#### Subtheme 3.3: Anxiety and fear elimination.

Adolescents described dramatic anxiety reductions following their participation in the 10-week MFGT program. The shift from hypervigilance and avoidance to social confidence enabled normal adolescent development and social participation, which participants directly attributed to the therapeutic benefits of MFGT.

“*Now I do not worry anymore knowing that if I take my drugs, I'll be fine I do not think about it again*.” (P10)

“*Initially, I do not go for dance practice anytime we were called because I used to think people would gossip about me, but now I go for dance practice, and I also perform*.” (P8)

These quotes suggest, in participants’ own accounts, that the MFGT intervention was associated with reduced pervasive anxiety and enabled participants to reclaim normative adolescent activities previously abandoned due to HIV-related fear. P10's description of reduced rumination (“I do not think about it again”) suggests, in her account, cognitive relief from the constant HIV preoccupation that had characterized her pre-intervention mental state.

P8's progression from social avoidance to active performance illustrates how reduced anxiety, in this account, extended beyond internal psychological states to behavioral change and social reintegration. This account suggests the intervention may have contributed to change not only in psychological symptoms but also in their social and developmental consequences, for this participant, enabling her to resume age-appropriate social roles and experiences

#### Subtheme 3.4: Psychological peace and well-being.

Beyond symptom reduction, adolescents described what they characterized as positive psychological states (peace of mind, contentment, happiness) which they associated with the MFGT intervention, a wellbeing they described as extending, in their accounts, beyond symptom management alone.

Participants described profound peace and positive well-being

“*I do not even have the time to be thinking about me having the virus or worrying about it. I am very okay my mind is at peace now*.” (P15)

“*Knowing that I'd be able to live long like a normal person makes me feel very okay*.” (P2)

These quotes demonstrate that the MFGT intervention achieved more than symptom amelioration; it facilitated positive mental health characterized by peace, contentment, and future-oriented hope. Together, these accounts reveal that the intervention successfully moved participants along the mental health continuum from crisis and dysfunction to genuine flourishing, characterized by peace, hope, normalcy, and freedom from HIV-related preoccupation, outcomes that extend far beyond traditional symptom reduction goals and approach what might be termed psychological thriving despite chronic illness.

## Discussion

This study documents participants’ accounts of a profound mental health crisis among adolescents living with HIV in Ghana, characterized by active suicidal ideation with method-specific planning, emotional dysregulation, and existential foreclosure. While prior research established that 25–44% of ALHIV experience suicidal ideation [1,2], our findings describe a progression from passive death wishes to active environmental assessment and alternative method-seeking in at least one participant's account, a critical distinction indicating imminent suicide risk [68]. The documented participant-reported reduction in suicidal ideation following the 10-week core content sessions of MFGT intervention represents a notable contribution to the sparse qualitative evidence base on family-based mental health interventions in resource-limited settings [38].

The convergence of parental abuse, classroom stigma triggers, and familial isolation in these accounts illuminates how environments participants expected to be protective paradoxically became, in their descriptions, trauma sources. We interpret this pattern, extending Pantelic et al.’s [17] work, as potentially reflecting stigma experienced as familial betrayal rather than merely external discrimination (an interpretive framing we offer cautiously, given it draws primarily on P3's single, detailed account rather than a pattern independently corroborated across multiple participants). P3's experience of diagnosis-triggered suicidal ideation compounded by parental verbal abuse is one we describe, as a researcher-generated interpretive label rather than a participant-derived term, using the phrase “compounded developmental trauma” to describe a pattern in which adolescents simultaneously process HIV diagnosis, developmental challenges, and attachment figure abuse [69]. This single case reveals a possible gap in current HIV care models (focusing on adolescent-level interventions while ignoring potentially undermining familial dynamics [23]), though this interpretation would benefit from corroboration across a larger sample. Similarly, P12's hypervigilance during classroom HIV education is consistent with, though it alone cannot establish, the possibility that well-intentioned public health messaging can traumatize HIV-positive students, highlighting a possible need for further examination of trauma-informed approaches in high-prevalence settings [70].

The transformation mechanisms participants described (cognitive reframing, universality, hope restoration, and emotional catharsis) represent participant-perceived contributors to change rather than empirically demonstrated causal mechanisms, since no experimental or dismantling design was used to isolate their individual effects; with that caveat, these perceived mechanisms align with group therapy theory [71] while demonstrating culturally specific applications. Our findings extend Yalom's [10] universality concept by revealing dual-level operation, horizontal (peer) and vertical (intergenerational family). P11's transformation upon discovering her mother and aunties also take antiretrovirals illustrates how family-level disclosure normalized HIV within kinship systems defining Ghanaian identity [19]. Beyond group therapy theory, these family-level findings also speak to the family systems and Structural Family Therapy premises underpinning MFGT's design [43]: P11's reframing occurred not through peer contact alone but through renegotiated family roles and disclosure patterns within her own household, consistent with the view that individual symptoms are embedded in, and altered through, family structure and communication rather than the individual in isolation. The cognitive reframing from “death sentence” to “manageable chronic illness like malaria” represents collective meaning-making through group process [72], distinguished from individual CBT by leveraging social proof [73]. Culturally resonant metaphors (malaria) rather than Western frameworks enhanced acceptability and effectiveness [50].

U = U education and prevention of mother-to-child transmission information were described by participants as reversing existential foreclosure, restoring marriage and parenthood aspirations (P5 specifically regarding marriage and sexual intimacy, and P4 regarding childbirth and fear of transmitting HIV to a future child) [74]. This unique contribution links biomedical advances to psychological outcomes, suggesting accurate treatment information functioned, in participants’ accounts, as a mental health intervention for ALHIV [75]. However, P3's pre-intervention crisis despite standard HIV counseling reveals information insufficiency without therapeutic processing, peer support, and family involvement [76]. MFGT's integration of biomedical education within therapeutic group context, reinforced through peer testimonials, family discussions, and emotional processing, may help explain superior effectiveness over standard post-test counseling, though this comparison was not directly tested in the present study [40].

The transformation from active suicidal crisis to “mind is at peace” represents outcomes rarely achieved in adolescent mental health interventions, particularly in resource-limited settings [26]. Participants’ narrated shifts in suicidal ideation, anxiety, emotional dysregulation, and HIV-related preoccupation were broader in scope than most outcomes reported in adolescent HIV literature [8]; suggesting that MFGT's simultaneous multi-level engagement (individual, peer, family) may plausibly have contributed to mutually reinforcing effects, although the absence of a comparison group means this cannot be attributed specifically to multi-level engagement versus single-level interventions [77]. The intergenerational format appeared, from participants’ accounts, to have ensured consistent messaging across all social contexts, potentially reducing cognitive dissonance when adolescents return to unsupportive environments [36]. Parental verbal abuse contributing to P3's crisis underscores why individual counseling alone cannot fully address distress rooted in family dysfunction [44], consistent with family systems theory's premise that individual symptoms are maintained by, and therefore require intervention within, the broader family system rather than the individual alone [43], challenging dominant individual-focused paradigms and supporting family-based approaches for relational and ecological mental health conceptualization in collectivist cultures [78].

### Candidate active ingredients of the intervention

Although this study cannot isolate the causal contribution of individual intervention components, participants’ accounts suggest several candidate active ingredients. Psychoeducation (particularly U = U and prevention-of-transmission content) appeared closely tied to hope restoration and reduced existential foreclosure; peer and family universality appeared closely tied to reduced isolation and shame; explicit mental health psychoeducation (e.g., the stress-rumination-suicide link) appeared tied to reduced rumination and greater help-seeking; and culturally resonant catharsis activities (drumming, singing, prayer) appeared tied to emotional regulation. These associations are drawn from participants’ own attributions of what helped them, rather than from a formal mechanism-evaluation design, and should be treated as hypotheses for future dismantling studies rather than confirmed active ingredients.

Situating these findings within the African family-based HIV mental health literature, our results broadly align with, while also extending, prior work such as the VUKA Family Program in South Africa, CHAMP-related family strengthening interventions, and Group Support Psychotherapy studies among adolescents in Uganda, all of which similarly report family-level engagement as a valuable lever for adolescent HIV-related mental health and psychosocial outcomes. Compared to this literature, the present study's qualitative focus on a period of acute suicidal crisis, and its documentation of participant-narrated shifts in suicidal ideation specifically, is a less commonly reported outcome in this evidence base, though differences in study design, outcome measurement, and population between these prior trials and the present qualitative process evaluation limit direct comparison.

### Implications for practice, policy and research

Practice Implications: Participants’ reports that culturally adapted family-based group therapy was associated with reduced suicidal ideation among adolescents who described active suicidal crisis suggest that task-shifted interventions delivered by trained lay counselors may be a promising avenue for mental health support without psychiatric consultation, pending confirmation through controlled research. This supports exploring the integration of evidence-based mental health interventions into existing HIV care platforms. Critical gaps in standard care are suggested by these accounts: adolescents described reaching crisis-level suicidal ideation following diagnosis, a pattern warranting further attention given current post-test counseling may be insufficient on its own. Mental health screening and proactive family-based intervention should be integrated into HIV diagnosis protocols rather than offered reactively when crisis develops. The finding that parental abuse compounded suicidal risk underscores why individual adolescent counseling cannot fully address distress rooted in family dysfunction, necessitating family-centered approaches.

Policy Implications: These findings challenge individual-focused paradigms, demonstrating mental health as fundamentally relational and ecological for ALHIV. Policymakers should prioritize family-based group interventions over resource-intensive individual counseling, serving multiple individuals simultaneously while addressing systemic factors maintaining distress. National HIV policies should mandate mental health integration into HIV care platforms with protocols for family-based interventions at diagnosis, treatment initiation, and transition points. Resource allocation should reflect cost-effectiveness of prevention versus reactive crisis management. Policies should support task-shifting through standardized training curricula and supervision structures.

Research Implications: Future research should examine durability of mental health gains through longitudinal follow-up, as current findings reflect immediate post-intervention outcomes, collected at a point when enthusiasm, emotional arousal, and recency effects may have shaped how participants described their experiences. Dismantling studies identifying necessary versus sufficient MFGT components would enable intervention optimization. Examining whether MFGT prevents mental health crisis when delivered proactively at diagnosis could inform prevention-focused implementation. Additional priorities include cost-effectiveness analyses, implementation science examining integration barriers, moderator analyses identifying who requires additional interventions, and investigation of impacts on adherence, viral suppression, and physical health outcomes.

### Limitations

This study has several limitations. First, the findings reflect immediate post-intervention outcomes, without longitudinal follow-up to assess the durability of mental health gains; interviews conducted immediately after intervention completion may have captured heightened enthusiasm, emotional arousal, and recency effects rather than stable, durable change. Second, the parent trial randomised only two clusters (one health facility per arm), and the small sample size limits generalizability beyond the study context; with only one cluster per arm, cluster-level confounding (e.g., site-specific staffing or contextual differences) cannot be distinguished from intervention effects in the parent trial, and our qualitative findings are similarly drawn from a single intervention-arm facility, though thematic saturation was achieved. Third, all participants received the MFGT intervention; absence of a comparison group prevents attribution of changes solely to the intervention versus other factors such as natural recovery or routine HIV care. Fourth, reliance on self-reported mental health changes without standardized psychometric assessment may introduce recall bias and limit comparability with quantitative studies. Fifth, the hospital-based recruitment may have excluded the most vulnerable adolescents who were disengaged from care, potentially limiting understanding of intervention effectiveness for those at highest risk. Sixth, participants were sampled exclusively from intervention completers who attended at least 7 of 10 core content sessions and volunteered for focus groups; this likely excluded ALHIV who dropped out, were dissatisfied with the intervention, or benefited least from it, meaning our findings may disproportionately reflect positive experiences and should not be read as representative of all ALHIV who began the MFGT program. Finally, as a qualitative study exploring lived experiences, findings provide rich descriptive insights into transformation mechanisms but cannot establish causal relationships or intervention efficacy; quantitative evaluation is needed to complement these qualitative findings.

## Conclusion

This study documents participants’ accounts of a profound mental health crisis among Ghanaian adolescents living with HIV, characterized by active suicidal ideation with method-specific planning. Participant-reported psychological transformation following culturally adapted 10 core content sessions of the- family-based group intervention. This suggests that task-shifted interventions may hold promise for resource-limited settings, though this qualitative study cannot establish suicide prevention or intervention efficacy in a formal sense. Key participant-perceived therapeutic mechanisms, dual-level universality, collective cognitive reframing through culturally resonant metaphors, and biomedical knowledge as psychological intervention, highlight the potential value of family-based approaches in collectivist contexts. These findings are consistent with challengeing individual-focused paradigms, suggesting mental health may be fundamentally relational and ecological for ALHIV. A researcher-generated interpretive lens we have termed “compounded developmental trauma” points toward a possible gap in care models that may ignore family dynamics, warranting further investigation. Exploring the integration of mental health screening and family-based interventions into HIV diagnosis protocols warrants consideration. Participants’ own accounts of transformation from suicidal crisis to psychological peace suggest that hope and healing may be achievable for ALHIV through ecologically informed interventions, warranting confirmation through further research.

## Supporting information

S1 Checklist COREQ Checklist. (DOCX)

S2 Checklist COSORT 25 Checklist. (DOCX)

S1 Text FGD Guide. (DOCX)
