# Supplementary material for: From suicidal ideation to hope: Perceived mental health transformation following a culturally adapted multiple family group therapy for adolescents living with HIV in Ghana - A qualitative process evaluation
Source: PLOS Ment Health. 2026 Nov 10;3(11):e0000600. doi: 10.1371/journal.pmen.0000600 (PMC13750614; doi:10.1371/journal.pmen.0000600)
Supplement: Supplementary file 3 [file pmen.0000600.s003.docx]

**FOCUS GROUP DISCUSSION GUIDE**

**Mental Health Transformation Among Adolescents Living with HIV**

*Following Culturally Adapted Multiple Family Group Therapy (MFGT)*

*Lower Manya Krobo District, Eastern Region, Ghana | NCT06701942*

**Principal Investigator:** Dorothy Serwaa Boakye

**Participants**: ALHIV aged 13–22 years who completed ≥7 of 10 MFGT sessions

**FGD Duration**: 60–90 minutes | 3 FGDs planned | n = 6–7 per group

**SECTION 1: WARM-UP QUESTION**

"To begin, could each person share your first name, age, gender, level of education, occupation, duration of HIV diagnosis, your primary caregiver and one word that describes how you are feeling today?"

**SECTION 2: MAIN DISCUSSION QUESTIONS**

**Domain A: Pre-intervention Mental Health Experiences (Question 1-3 )**

**Purpose**: To explore mental health challenges ALHIV experienced prior to MFGT, including suicidal ideation, emotional distress, and contextual risk factors.

Q1. How would you describe your emotional and mental state before you started the 'Abusuafoɔ Apomuden' sessions?

**Probe**: What kinds of thoughts were going through your mind when you first learned about your HIV status?

**Probe**: Were there times when you felt like you did not want to continue living or thought about harming yourself? (Respond sensitively; counsellor on standby.)

**Probe**: How did those feelings affect your daily life at home, at school, or with friends?

Q2. Were there things happening around you at home, at school, or within your family that made your mental health worse at that time?

**Probe**: How did family members respond when HIV was discussed?

**Probe**: Did anything at school such as HIV lessons affect how you felt about yourself?

Q3. Before joining this programme, had you ever spoken to anyone about these feelings? If not, what prevented you?

**Probe:** Were there people you felt you could not talk to? Why?

**DOMAIN B	Mechanisms of Psychological Change (Questions 4–9)**

**Purpose**: To identify the specific therapeutic mechanisms participants perceived as facilitating mental health change during the MFGT programme. This is the primary focus of the study.

Q4. What was it like when you first started attending the sessions? what were you expecting, and what did you find?

**Probe**: How did it feel to be in a group with other families who are also living with HIV?

Q5. Which parts of the programme had the biggest impact on the way you were thinking or feeling about yourself and your HIV status?

**Probe**: Can you describe what was taught or discussed in those sessions?

**Probe**: Were there particular sessions, activities, or conversations that stand out in your memory?

Q6. During the programme you learnt that HIV is undetectable and untransmittable when you take your medicines consistently. What effect, if any, did that information have on you?

**Probe**: Did this knowledge change how you see your future? In what way?

Q7. How did being in the group with other adolescents who are also living with HIV affect you personally?

**Probe**: Did seeing others living with HIV change the way you thought about yourself or your situation?

**Probe**: Were there specific things group members said or did that helped you?

Q8. How did the involvement of your family caregiver in the programme affect you?

**Probe**: Were there moments during the sessions that were difficult between you and your caregiver?

Q9. How did the cultural activities such as drumming, singing, dancing, and prayer make you feel during the sessions?

**Probe**: Were there moments when those activities helped you release emotions you had been carrying?

**DOMAIN C: Post-Intervention Mental Health Outcomes (Questions 10–11)**

**Purpose**: To document the mental health transformations participants experienced following MFGT, including resolution of suicidal ideation, emotional regulation, and psychological wellbeing.

Q10. Comparing how you felt before the programme to how you feel now — what has changed for you, mentally and emotionally?

**Probe:** Do you still have thoughts of harming yourself, or has that changed?

**Probe**: How do you manage your emotions now when things are difficult?

Q11. How would you describe your peace of mind since completing the programme? What words come to mind when you think about your life and your future now?

**Probe**: Do you feel hopeful? What gives you that hope?

**Probe:** Is there anything about your mental health that you feel still needs attention or support?

**DOMAIN D:	Acceptability and Recommendations (Question 12)**

Purpose: To gather participants' views on the cultural appropriateness and acceptability of the intervention, and to elicit suggestions for improvement.

Q12. If you were advising another young person living with HIV about this programme, what would you tell them? And if you could change or add anything to it, what would that be?
