# Supplementary material for: From suicidal ideation to hope: Perceived mental health transformation following a culturally adapted multiple family group therapy for adolescents living with HIV in Ghana - A qualitative process evaluation
Source: PLOS Ment Health. 2026 Nov 10;3(11):e0000600. doi: 10.1371/journal.pmen.0000600 (PMC13750614; doi:10.1371/journal.pmen.0000600)
Supplement: Supplementary file 4 [file pmen.0000600.s005.docx]

**Response to Reviewers**

*Manuscript PMEN-D-26-00136 – From Suicidal Ideation to Hope: Perceived Mental Health Transformation Following a Culturally Adapted Multiple Family Group Therapy for Adolescents Living with HIV in Ghana — A Qualitative Process Evaluation*

We thank the editor and all three reviewers for their careful and constructive reading of our manuscript. Below we provide a point-by-point response to each comment. Reviewer comments are reproduced in italics, followed by our response. Corresponding changes have been made throughout the revised manuscript and are marked in red text for ease of review.

# **Reviewer 1**

**Comment 1:** *The Abstract mentions three major themes but appears to list four.*

**Response:** Thank you for catching this ambiguity. We have revised the Results sentence in the Abstract to explicitly enumerate the three overarching themes by name: (1) Pre-Intervention Mental Health Crisis, (2) Psychological Change Mechanisms, and (3) Mental Health Transformation, each of which encompasses several subthemes. This should remove any impression that four themes were reported. Page 1-2

**Comment 2:** *The Introduction refers to “implementation constraints of resource-limited settings” being addressed by MFGT, but no such constraints were described beforehand.*

**Response:** We have revised the paragraph preceding this statement to explicitly name the workforce shortages and infrastructural gaps (e.g., fewer than two psychiatrists per million population, limited psychologists trained in evidence-based psychotherapies) as “the core implementation constraints,” and we now explicitly reference these constraints when introducing MFGT (“Given the workforce shortages and infrastructural gaps described above…”), so the connection is explicit rather than assumed. Page 3

**Comment 3:** *How is “mental health transformation” described/operationalised in the Introduction?*

**Response:** We have added an explicit operational definition in the Introduction: mental health transformation is defined as participant-narrated shifts in psychological state and subjective wellbeing (suicidal ideation, emotional regulation, anxiety, and hope) as expressed in participants’ own accounts, rather than as clinically diagnosed or biomarker-verified change. This definition is now stated before the study aim. Page 5

**Comment 4:** *What systematic frameworks guided the cultural adaptation? The manuscript states the full adaptation process is “described elsewhere” without a citation.*

**Response:** We have named the specific frameworks that guided the cultural adaptation directly in the Methods: the Theory of Triadic Influence, Social Action Theory, and the Stirman et al. adaptation coding framework guided the adaptation phase specifically, with family systems theory guiding pilot implementation and the RE-AIM framework guiding the evaluation phase (as now described in the Study Design subsection). We have also added substantially more detail on the four-phase community stakeholder engagement process itself, including its timeline (September 2024 to February 2025) and the composition of the 34 community stakeholders involved (caregivers, older family members, adolescents and young adults, family heads, healthcare professionals, and Community Health Officers). A citation for the separate methodological paper describing the full adaptation process in detail has also been added (reference 58); the exact citation details for this reference require the authors’ confirmation before submission. In the course of this revision we also identified and corrected an inconsistency in the original manuscript, where the in-text citation for the source 4Rs and 2Ss model incorrectly read “McKay et al., 2011” despite the reference list correctly citing McKay et al. (1995); this has been corrected to cite the reference list entry directly. Page 7

**Comment 5:** *Please provide a citation for QDA Miner.*

**Response:** A software citation has been added in the Data Analysis subsection (Provalis Research, Montreal, QC, Canada; reference 63). The exact version number and year of use should be confirmed by the authors before submission, as we were not able to verify this detail independently. Page 11

**Comment 6:** *Does “diagnosed with HIV for 12–15 years” (Table 1) mean participants were born with HIV or acquired it after birth?*

**Response:** We have added a clarifying sentence to the narrative accompanying Table 1: the majority of participants with 12–15 years since diagnosis acquired HIV perinatally, while other participants acquired HIV later through sexual transmission. Page 13

**Comment 7:** *Several subthemes (e.g., 2.3) are supported by only one participant quote, yet the text says “these quotes demonstrate…”, which is misleading. There is also overrepresentation of P3 (6 times) relative to other participants (P2, P5 at 3 times each) out of 19 participants.*

**Response:** We revisited the original focus group transcripts and identified additional, previously unused illustrative quotes from other participants for each of the single-quote subthemes: Subtheme 2.3 (Hope Restoration) now includes a second quote (P4) on childbirth/PMTCT alongside the existing marriage quote; Subtheme 2.4 (Psychoeducation on Mental Health) now includes a second quote (P16) on help-seeking; Subtheme 2.5 (Emotional Catharsis) now includes two additional quotes (P8 on prayer, P19 on singing) alongside the existing drumming quote; and Subtheme 1.3 (Risk Factors) now includes an additional caregiver-abuse quote (P17, grandmother) alongside the existing father-abuse quote from P3, reducing reliance on a single participant for this pattern. The accompanying interpretive text for each of these subthemes has been revised to reflect and integrate the additional quotes rather than referring to “these quotes” when only one was originally presented. Page 14-24

**Comment 8:** *The Discussion states that U=U education “restored marriage and parenthood aspirations,” but no participant quote in the Results mentions parenthood aspirations specifically.*

**Response:** This concern is now resolved by the addition described above: the Results (Subtheme 2.3) include a specific quote from P4 regarding childbirth and fear of transmitting HIV to a future child. The Discussion paragraph has been revised to explicitly attribute the marriage/intimacy aspect to P5 and the parenthood/childbirth aspect to P4, so the claim is now traceable to specific participant data. Page 19

# **Reviewer 2**

**Comment 1:** *The claim that “few evidence-based family interventions for ALHIV mental health have been developed and tested in African settings” needs qualification; relevant work exists (Ssewamala and colleagues in Uganda/Kenya; the VUKA family program in South Africa; prior MFG work in Ghana).*

**Response:** We have revised this paragraph to explicitly acknowledge this existing body of work, citing Ssewamala’s Uganda family-strengthening/economic-support interventions and the VUKA family program in South Africa. We have narrowed the stated gap to be more specific: culturally adapted Multiple Family Group Therapy models targeting suicidal ideation and severe mental health crisis among ALHIV, evaluated through in-depth qualitative process evaluation within a Ghanaian context, which remain comparatively scarce. Page 4

**Comment 2:** *References 27 and 28 appear in the reference list but are not cited in the body of the manuscript; reference 28 (Bhana) is particularly relevant to family-based interventions.*

**Response:** Both references are now cited in the Introduction: reference 27 (Woollett; trauma-informed care now cited as 28) and reference 28 (Bhana systematic review now cited as 29) are cited together to support the statement regarding the paucity of trauma-informed mental health interventions for ALHIV in LMICs, and reference 28 is cited again in the paragraph on family-based interventions in Africa. Page 3

**Comment 3:** *The statement that “MFGT has demonstrated effectiveness for adolescent behavioral health challenges in high-income countries” should be revised to acknowledge sub-Saharan African evidence, given the study setting.*

**Response:** This sentence has been revised to state that MFGT and related family-based group models have demonstrated effectiveness in high-income countries and, increasingly, in sub-Saharan African settings, with reference to family-strengthening interventions in Uganda and the VUKA family program in South Africa. Page 4

**Comment 4:** *The Introduction could more directly link family functioning to suicidal ideation.*

**Response:** We have added a sentence in the Introduction explicitly stating that family conflict and poor caregiver-adolescent communication have been directly linked to elevated suicidal ideation among ALHIV, positioning family functioning as both a risk factor for, and a potential lever against, adolescent suicidality. Page 3

**Comment 5:** *The theoretical framing needs clarification: the manuscript cites family systems theory broadly but references Minuchin, who is more specifically associated with Structural Family Therapy.*

**Response:** We have revised the relevant sentence to clarify that MFGT is grounded broadly in family systems theory while also drawing specifically on Minuchin’s Structural Family Therapy for its emphasis on family structure, roles, hierarchy, and communication patterns, and we briefly explain how this structural lens informed the study’s attention to family roles and communication patterns as both contributors to, and pathways of recovery from, adolescent mental health crisis. Page 4

**Comment 6:** *Similar MFG models have previously been adapted in African settings (e.g., McKay and colleagues’ work in Uganda/Ghana); the adaptation pathway for the Ghanaian version used in this study should be clarified – was it adapted directly from the U.S. model, from a Ugandan adaptation, from prior Ghanaian MFG work, or a combination?*

**Response:** We have clarified in the Methods that Abusuafoɔ Apomuden was adapted directly from the original U.S. 4Rs and 2Ss model/version (McKay et al., 1995), organised around the model’s core components (Rules, Responsibility, Relationships, and Respectful communication; Stress and Social support), rather than through an intermediate regional adaptation. We have substantially expanded this description to include the specific theoretical frameworks that guided the adaptation (Theory of Triadic Influence, Social Action Theory, and the Stirman et al. adaptation coding framework), the structured four-phase stakeholder engagement process and its timeline (September 2024–February 2025), and the composition of the 34 stakeholders involved. We have also added a citation to a separate methodological paper describing this adaptation process in full detail (reference 68), and, for comparison, we now note that the group sizes used are consistent with those of the South African VUKA and Ugandan Amaka Amasanyufu adaptations of the same 4Rs and 2Ss model. Page 7-8

**Comment 7:** *The statements “Participants were ALHIV who completed the 10-week MFGT intervention” and “all ALHIV who completed at least 7 of the 10 MFGT sessions were eligible” are inconsistent.*

**Response:** This inconsistency has been resolved. The Participants and Recruitment subsection now consistently states that participants had completed at least 7 of the 10 MFGT sessions, the threshold used to define adequate intervention exposure, removing the earlier implication that full completion was required. Page 9

**Comment 8:** *How were the 19 participants selected, and are there intervention/control arms in the parent trial?*

**Response:** We have clarified that the parent trial is a two-arm cluster randomized controlled trial with an MFGT intervention arm and a standard-of-care control arm, and that participants for this qualitative component were drawn exclusively from the intervention arm (purposively sampled from those meeting the 7-of-10-session attendance threshold), as the focus group discussions were designed specifically to explore experiences of MFGT participation rather than to compare arms. Page 9

**Comment 9:** *Discussion language sometimes implies causality despite the qualitative, post-intervention-only design (e.g., “created synergistic effects unattainable through single-level interventions”).*

**Response:** We have revised this specific sentence and surrounding passages to acknowledge that, in the absence of a comparison group, any apparent synergistic effect cannot be attributed specifically to multi-level engagement versus single-level interventions, and we have softened related causal language throughout the Discussion (e.g., “may plausibly have contributed to,” “appeared, from participants’ accounts, to…”). Page 24-26

**Comment 10:** *Since MFGT is grounded in family systems theory as well as group therapy theory, the Discussion should explain findings in relation to family systems theory, not only group therapy theory.*

**Response:** We have added a new passage to the Discussion explicitly connecting the family-level findings (P11’s account of discovering her mother and aunties also take antiretrovirals) to family systems theory and Structural Family Therapy premises, distinguishing this from the group therapy theory discussion that precedes it. Page 25

# **Reviewer 3**

**Comment 1:** *The title does not indicate the study design, and phrases such as “From Suicidal Ideation to Hope,” “Mental Health Transformation,” and “Through a Culturally Adapted MFGT” imply a causal relationship that the qualitative design cannot support.*

**Response:** The subtitle already identified the design (“A Qualitative Process Evaluation”); we have retained this. To address the causal-language concern while preserving the paper’s narrative framing, we have revised the title to “From Suicidal Ideation to Hope: Perceived Mental Health Transformation Following a Culturally Adapted Multiple Family Group Therapy for Adolescents Living with HIV in Ghana — A Qualitative Process Evaluation,” replacing “Through” (which implies a causal mechanism) with “Following,” adding “Perceived” to signal that transformation is participant-reported, and shortening the title overall (from 35 to 28 words) for concision. Page 1

**Comment 2:** *The Abstract contains overstated phrases such as “complete mental health transformation” and “suicidal ideation cessation.”*

**Response:** These phrases have been softened throughout the Abstract. “Complete mental health transformation” is now “substantial perceived improvements in mental health,” and “suicidal ideation cessation” and “anxiety elimination” are now “reduced suicidal ideation” and “reduced anxiety,” respectively. The Aim statement has also been revised to specify “ALHIV’s perceived mental health transformation.” Page 1

**Comment 3:** *The parent trial is insufficiently described (design, arms, participant flow, theoretical framework, primary outcomes, current status).*

**Response:** We have substantially expanded the Study Design and Setting subsection to describe the parent trial in detail: it is a two-arm, cluster-randomised controlled trial in which the health facility (clinic) served as the unit of randomisation, evaluating the effects of the intervention on depressive symptoms, anxiety symptoms, and antiretroviral therapy (ART) adherence among young people living with HIV and their caregivers. Atua Government Hospital was allocated to the intervention arm and Asesewa Government Health Facility to the control arm using a computer-generated randomisation sequence, with allocation concealment maintained by withholding clinic allocation from research team members conducting baseline assessments until enrolment was complete at both sites; randomisation and allocation were finalised by 31 May 2025. We now explicitly note that, as only two clusters (one per arm) were randomised, the design provides no within-arm cluster replication, a constraint discussed further in the Limitations. Primary outcomes (depression, anxiety, ART adherence) and the instruments used (PHQ-9, GAD-7, a validated adherence measure) are specified, along with the trial’s theoretical grounding across phases (Theory of Triadic Influence, Social Action Theory, and Stirman’s adaptation framework guiding adaptation; family systems theory guiding pilot implementation; the RE-AIM framework guiding evaluation), its prospective registration date relative to enrolment, and its current status (data collection completed, analysis in progress, with quantitative findings to be reported separately). In the course of adding this detail we also identified and corrected an internal inconsistency: the Limitations had described a “single-site” design, which was inconsistent with the two-cluster (two-facility) design now described; this has been corrected, and a new limitation has been added noting that with only one cluster per arm, site-level confounding cannot be distinguished from intervention effects. Page 5-6

**Comment 4:** *The intervention is described only briefly; the authors should use the TIDieR checklist.*

**Response:** Rather than presenting a separate TIDieR-formatted summary alongside the narrative intervention description, we have integrated all TIDieR checklist domains (rationale, materials, procedures, providers, mode and setting of delivery, dose, tailoring, and fidelity) directly into the narrative description of the intervention, with an opening sentence explicitly stating that the description follows the TIDieR (Template for Intervention Description and Replication) checklist and citing the TIDieR guideline (reference 54). This avoids redundancy between a structured summary and the narrative account while still ensuring every TIDieR domain is addressed, including intervention materials (facilitator manuals, session guides, psychoeducational materials, take-home assignments), mode of delivery (face-to-face, in-person), and tailoring (two modules receiving additional reinforcement across cohorts in response to attendance and family-conflict patterns observed during delivery). Page 7-8

**Comment 5:** *The rationale for interviewing only intervention-arm participants (with no control-arm comparison) should be explained.*

**Response:** We have added an explicit statement in the Participants and Recruitment subsection clarifying that this qualitative process evaluation was scoped and funded specifically to explore ALHIV’s and caregivers’ experiences of MFGT participation and to surface participant-perceived mechanisms of change, and was not designed to establish comparative effectiveness against the control arm, which is being assessed through the parent trial’s quantitative outcome analysis. We have also added this as an explicit limitation in the Discussion, acknowledging that without control-arm data we cannot determine whether reported changes are specific to MFGT or reflect non-specific therapeutic factors (hope, attention, group belonging) common to supportive interventions generally. Page 9

**Comment 6:** *More information is needed on family group composition – who attended each session, whether caregivers were involved throughout, and how family interaction was incorporated.*

**Response:** We have clarified in the Methods that each enrolled adolescent attended every session together with their participating caregiver; sessions were not split into separate adolescent-only or caregiver-only components, and family-level activities (joint role-plays, family discussions, shared therapeutic activities) were incorporated into every session. Page 7

**Comment 7:** *The manuscript should not conclude that suicidal ideation, anxiety, or emotional dysregulation were “eliminated” or that all participants achieved transformation, since only participants’ perceptions were assessed, without a comparison group or standardized instruments.*

**Response:** We have systematically revised the language throughout the Results (Theme 3 subthemes) and Discussion to remove absolute/universal claims (e.g., “all adolescents,” “complete cessation,” “elimination,” “across all participants”) and replaced them with hedged, participant-attributed language (e.g., “participants described,” “in their own accounts,” “this was not systematically assessed”). We now explicitly state, where relevant, that these are self-reported perceptions rather than clinically verified outcomes. Page 22-23 and from 24-26

**Comment 8:** *Sampling strategy requires clarification, as intervention completers who volunteered for FGDs likely exclude dropouts and dissatisfied participants, potentially biasing findings toward positive experiences.*

**Response:** We have added this as an explicit limitation: participants were sampled exclusively from intervention completers who attended at least 7 of 10 sessions and volunteered for focus groups, likely excluding adolescents who dropped out, were dissatisfied, or benefited least, meaning the findings may disproportionately reflect positive experiences and should not be read as representative of all ALHIV who began the MFGT programme. Page 29

**Comment 9:** *Figure 2 (“psychological change mechanism”) appears to imply an established causal pathway that cannot be supported by qualitative findings without a control group.*

**Response:** The in-text reference to Figure 2 has been revised to clarify that the figure summarizes psychological change mechanisms as perceived and reported by participants, and depicts a descriptive summary of participant accounts rather than an empirically established causal pathway, given the absence of a comparison group. The figure too has been revised to reflect this change. **See fig 2: supplementary file.**

**Comment 10:** *Concepts such as “compounded developmental trauma,” “existential foreclosure,” and “stigma as familial betrayal” appear researcher-generated rather than participant-derived, and the manuscript should distinguish participant experience, researcher interpretation, and theoretical explanation.*

**Response:** We have revised the relevant Discussion paragraph to explicitly frame “familial betrayal” and “compounded developmental trauma” as researcher-generated interpretive labels rather than participant-derived terms, note that the “familial betrayal” framing draws primarily on a single participant’s (P3’s) detailed account rather than a pattern independently corroborated across multiple participants, and state that these interpretations would benefit from corroboration across a larger sample. Similar hedging has been applied to the related claim about classroom stigma triggers (P12). Page 24-26

**Comment 11:** *The manuscript makes universal statements (e.g., “all adolescents who reported pre-intervention crying described complete cessation,” “complete resolution…across all participants”) without systematic verification.*

**Response:** As noted above, these statements have been revised throughout Theme 3 and the Discussion to remove universal claims and replace them with participant-attributed, hedged language, with explicit acknowledgment that no systematic assessment was conducted to verify uniform improvement across all participants. Page 21-24

**Comment 12:** *The Discussion would benefit from a more detailed examination of which intervention techniques (psychoeducation, peer support, family communication, normalization, emotional expression, etc.) may have driven perceived change – the “active ingredients” of the intervention – and should distinguish perceived mechanisms from empirically demonstrated mechanisms.*

**Response:** We have added a new “Active Ingredients” paragraph to the Discussion identifying candidate mechanisms drawn from participants’ own attributions (psychoeducation/U=U tied to hope restoration; peer and family universality tied to reduced isolation; mental health psychoeducation tied to reduced rumination and help-seeking; culturally resonant catharsis activities tied to emotional regulation), explicitly framed as hypotheses for future dismantling studies rather than confirmed active ingredients. We have also added an explicit hedge earlier in the Discussion clarifying that the documented mechanisms represent participant-perceived contributors to change rather than empirically demonstrated causal mechanisms, since no experimental or dismantling design was used to isolate their individual effects. Page 27

**Comment 13:** *The Discussion should be strengthened by comparison with other African family- and group-based HIV mental health interventions (VUKA, CHAMP, Family Strengthening Intervention, Group Support Psychotherapy studies from Uganda, etc.).*

**Response:** We have added a new paragraph situating our findings within this literature, noting broad alignment with the VUKA Family Program (South Africa), CHAMP-related family strengthening interventions, and Group Support Psychotherapy studies among adolescents in Uganda, while noting that our qualitative focus on a period of acute suicidal crisis and participant-narrated shifts in suicidal ideation specifically is a less commonly reported outcome in this literature, with the caveat that direct comparison is limited by differences in study design, outcome measurement, and population. Page 24-26

**Comment 14:** *Immediate post-intervention interviews may capture enthusiasm, emotional arousal, and recency effects rather than durable change; this should be discussed as a limitation.*

**Response:** We have added this explicitly to both the Discussion (Research Implications) and the Limitations section, noting that interviews conducted immediately after intervention completion may have captured heightened enthusiasm, emotional arousal, and recency effects rather than stable, durable change, and that longitudinal follow-up is needed to determine whether reported changes persist. Page 28-29

**Comment 15:** *The assertion that “thematic saturation was achieved with 19 participants” requires stronger justification given the breadth of themes (suicidality, family relationships, stigma, hope, adherence, mental health).*

**Response:** We have strengthened the Data Saturation subsection to specify the coding framework used (an inductively developed codebook applied consistently by two coders), how saturation was monitored (structured team debriefs after each FGD comparing new codes against the existing codebook), and we now explicitly note that saturation is interpreted as domain-specific – achieved for the recurring patterns reported – rather than as an exhaustive account of every possible theme within this population, given the breadth of topics covered. Page 9

**Comment 16:** *Minor: Trial registration occurred in November 2024; clarify whether this was prospective relative to enrollment.*

**Response:** We have added a sentence confirming that the trial was prospectively registered on ClinicalTrials.gov (NCT06701942) on 22 November 2024, prior to participant enrollment commencing on 10 December 2024. Page 6

**Comment 17:** *Minor: Participants include individuals up to age 22; explain why they are classified as “adolescents.”*

**Response:** We have added a citation to the WHO/UNAIDS extended definition of adolescents and young people (encompassing ages 10–24 for HIV programming and research purposes) alongside the eligibility criteria to justify this age range classification. Page 9
