# Supplementary material for: From suicidal ideation to hope: Perceived mental health transformation following a culturally adapted multiple family group therapy for adolescents living with HIV in Ghana - A qualitative process evaluation
Source: PLOS Ment Health. 2026 Nov 10;3(11):e0000600. doi: 10.1371/journal.pmen.0000600 (PMC13750614; doi:10.1371/journal.pmen.0000600)
Supplement: Supplementary file 5 [file pmen.0000600.s006.docx]

**Response to the Reviewer’s comment**

**Comment 1:** *Figures 1 and 2 are blurred in the material provided, and I cannot read their contents. Please ask the authors to supply clear, high-resolution versions of both figures.*

**Response:** We thank the reviewer for flagging this. Both figures have been rebuilt from vector source files and re-exported as 600 DPI, RGB TIFF files (Figure 1: 9000 × 5210 px; Figure 2: 7087 × 6462 px), replacing the previously submitted SVG versions that evidently did not render clearly in the reviewer's materials. Figure 1 (Qualitative Component Flow Diagram) and Figure 2 (Participant-Reported Themes in Perceived Psychological Change Following MFGT Participation) are both resubmitted in this format.

**Comment 2:** *The data availability statement says that the data are in the manuscript or a supplementary file, but I cannot find a link to the underlying data. Please ask the authors to provide a repository link to the de-identified raw data and update the statement accordingly.*

**Response:** We have deposited de-identified sample focus group discussion transcripts, the focus group discussion guide, the codebook, the thematic framework, and the aggregated sociodemographic summary dataset underlying Table 1 at Mendeley Data (<https://doi.org/10.17632/25xyfbhkf4.1>), and have updated the Data Availability Statement accordingly. The revised statement reads: "De-identified sample focus group discussion transcripts, the focus group discussion guide, the codebook, the thematic framework, and the aggregated sociodemographic summary dataset underlying Table 1 are available at Mendeley Data: <https://doi.org/10.17632/25xyfbhkf4.1>. Full raw transcripts are not publicly available, as the small focus group sizes, participants' HIV status, and the inclusion of minors create a meaningful risk of re-identification even after de-identification; qualified researchers may request access to full transcripts from the corresponding author, subject to institutional ethics approval and a data use agreement."
