# Supplementary material for: From suicidal ideation to hope: Perceived mental health transformation following a culturally adapted multiple family group therapy for adolescents living with HIV in Ghana - A qualitative process evaluation
Source: PLOS Ment Health. 2026 Nov 10;3(11):e0000600. doi: 10.1371/journal.pmen.0000600 (PMC13750614; doi:10.1371/journal.pmen.0000600)
Supplement: Peer review history

# Peer review: Decision Letter 0

**DOI:** 10.1371/journal.pmen.0000600.r001

**Authors:** Karli Montague-Cardoso

8 Jun 2026

PMEN-D-26-00136

From Suicidal Ideation to Hope: Mental Health Transformation Through a Culturally Adapted Multiple Family Group Therapy for Adolescents Living with HIV in Ghana

PLOS Mental Health

Dear Dr. Adjorlolo,

Thank you for submitting your manuscript to PLOS Mental Health. I am sorry for the delay in reaching a decision. This was due to difficulties securing reviewers. After careful consideration of the reviewer reports, which we have now received, we feel that your paper has merit but does not fully meet PLOS Mental Health’s publication criteria as it currently stands. Therefore, we invite you to submit a revised version of the manuscript that addresses the points raised during the review process. Please address all of the comments, which you can find below and in the attached document.

Please submit your revised manuscript by Aug 07 2026 11:59PM. If you will need more time than this to complete your revisions, please reply to this message or contact the journal office at mentalhealth@plos.org. When you're ready to submit your revision, log on to https://www.editorialmanager.com/pmen/ and select the 'Submissions Needing Revision' folder to locate your manuscript file.

Please include the following items when submitting your revised manuscript:

- A letter that responds to each point raised by the editor and reviewer(s). You should upload this letter as a separate file labeled 'Response to Reviewers'.
- A marked-up copy of your manuscript that highlights changes made to the original version. You should upload this as a separate file labeled 'Revised Manuscript with Track Changes'.
- An unmarked version of your revised paper without tracked changes. You should upload this as a separate file labeled 'Manuscript'.

Guidelines for resubmitting your figure files are available below the reviewer comments at the end of this letter.

As the corresponding author, your ORCID iD is verified in the submission system and will appear in the published article. PLOS supports the use of ORCID, and we encourage all coauthors to register for an ORCID iD and use it as well. Please encourage your coauthors to verify their ORCID iD within the submission system before final acceptance, as unverified ORCID iDs will not appear in the published article. *Only*  the individual author can complete the verification step; PLOS staff *cannot*  verify ORCID iDs on behalf of authors.

We look forward to receiving your revised manuscript.

Kind regards,

Karli Montague-Cardoso

Staff Editor

PLOS Mental Health

**Journal Requirements:**

1.  Please amend your detailed Financial Disclosure statement. This is published with the article. It must therefore be completed in full sentences and contain the exact wording you wish to be published.

i. Please clarify all sources of financial support for your study. List the grants, grant numbers, and organizations that funded your study, including funding received from your institution. Please note that suppliers of material support, including research materials, should be recognized in the Acknowledgements section rather than in the Financial Disclosure.

ii. State the initials, alongside each funding source, of each author to receive each grant. For example: "This work was supported by the National Institutes of Health (####### to AM; ###### to CJ) and the National Science Foundation (###### to AM)."

iii. State what role the funders took in the study. If the funders had no role in your study, please state: “The funders had no role in study design, data collection and analysis, decision to publish, or preparation of the manuscript.”

iv. If any authors received a salary from any of your funders, please state which authors and which funders.

2. We note that your Data Availability Statement is currently as follows: All data relating to this submission is available in the manuscript or as a supplementary file.

Please confirm at this time whether or not your submission contains all raw data required to replicate the results of your study. Authors must share the “minimal data set” for their submission. PLOS defines the minimal data set to consist of the data required to replicate all study findings reported in the article, as well as related metadata and methods (https://journals.plos.org/plosone/s/data-availability#loc-minimal-data-set-definition).

For example, authors should submit the following data:

- The values behind the means, standard deviations and other measures reported;

- The values used to build graphs;

- The points extracted from images for analysis.

Authors do not need to submit their entire data set if only a portion of the data was used in the reported study.

If your submission does not contain these data, please either upload them as Supporting Information files or deposit them to a stable, public repository and provide us with the relevant URLs, DOIs, or accession numbers. For a list of recommended repositories, please see https://journals.plos.org/plosone/s/recommended-repositories.

If there are ethical or legal restrictions on sharing a de-identified data set, please explain them in detail (e.g., data contain potentially sensitive information, data are owned by a third-party organization, etc.) and who has imposed them (e.g., an ethics committee). Please also provide contact information for a data access committee, ethics committee, or other institutional body to which data requests may be sent. If data are owned by a third party, please indicate how others may request data access.

3. Please ensure that your Ethics Statement is available in its entirety at the beginning of your Methods section, under a subheading 'Ethics Statement'.

4. Please provide separate figure files in .tif or .eps format.

For more information about figure files please see our guidelines:

https://journals.plos.org/mentalhealth/s/figures

https://journals.plos.org/mentalhealth/s/figures#loc-file-requirements

5. Please ensure that the Title in your manuscript file and the Title provided in your online submission form are the same.

6. Tables should not be uploaded as individual files. Please remove these files and include the Tables in your manuscript file as editable, cell-based objects. For more information about how to format tables, see our guidelines:

https://journals.plos.org/mentalhealth/s/tables

7. We have noticed that you have uploaded Supporting Information files, but you have not included a list of legends. Please add a full list of legends for your Supporting Information files after the references list.

If the reviewer comments include a recommendation to cite specific previously published works, please review and evaluate these publications to determine whether they are relevant and should be cited. There is no requirement to cite these works unless the editor has indicated otherwise.

**Additional Editor Comments (if provided):**

[Note: HTML markup is below. Please do not edit.]

Reviewers' comments:

Reviewer's Responses to Questions

**Comments to the Author**

1. Does this manuscript meet PLOS Mental Health’s publication criteria?

Reviewer #1: Yes

Reviewer #2: Yes

Reviewer #3: No

**********

2. Has the statistical analysis been performed appropriately and rigorously?

Reviewer #1: N/A

Reviewer #2: Yes

Reviewer #3: No

**********

3. Have the authors made all data underlying the findings in their manuscript fully available (please refer to the Data Availability Statement at the start of the manuscript PDF file)?

The PLOS Data policy

Reviewer #1: Yes

Reviewer #2: Yes

Reviewer #3: No

**********

4. Is the manuscript presented in an intelligible fashion and written in standard English?

Reviewer #1: Yes

Reviewer #2: Yes

Reviewer #3: No

**********

**Reviewer #1:**  Summary: This paper presents findings from a study about mental health transformation through a culturally adapted multiple family group therapy for adolescents living with HIV in Ghana. The study used a qualitative research design with 19 ALHIV and collected data through 3 FGDs. The findings revealed that participants experienced improvements in their mental health, had restored hope and the authors show the importance of dealing with mental health issues that adolescents face on the systemic level. I submit that this paper should be accepted after minor revisions.

Strengths of the paper: The study addresses a major public health issue; ALHIV suicidal ideation and mental health. The authors show the major gap that this study is filling, which is lack of culturally resonant therapy models to deal with suicidal ideation among ALHIV, with an additional component of the family. The study is qualitatively rigorous. The findings are explained in a way that is easily understood by the reader. Additionally, the authors provide the major contributions of the study and how the findings can be used to influence policy and practice in Ghana for the wellbeing of ALHIV.

Minor Revisions

Abstract (page 2) there is mention of three major themes that emerged but it seems the listed themes are four. Please check/clarify.

Introduction (page 4-line2) there is mention of implementation constraints of resource limited settings being addressed by MFGT and yet there are none mentioned before this. Please provide a brief description of the implementation constraints before this paragraph.

Introduction (page 4) How is mental health transformation described?

About the intervention (page 5); what are the systematic frameworks that were followed? Describe them or mention and cite them; Additionally, it is written that the full adaptation process is described elsewhere; please provide a citation for a paper where this description is given or provide the description here. This paper sets out to show how a culturally adapted MFGT for ALHIV transforms their mental health, there is need to show/cite/mention how the adaptation was done.

Data analysis (page 8); provide a citation for QDA Miner.

Findings (page 9); When you mention “Nearly half had been diagnosed with HIV for 12-15 years,” does this mean that they were born with it or did they acquire it after birth?

Subtheme 2.3; there is one participant excerpt talking about hope restoration, please provide more quotes or at least more than one to solidify the need for this subtheme to be included in the results. Please do the same for the other subthemes that have one quote. Additionally, when you present one quote and follow it with “these quotes demonstrate”, it is misleading to the reader. Additionally, there is an over representation of P3. Out of the 19 participants, 11 are represented and P3’s quotes appear 6 times, P2 and P5 3 times each. Given that data was saturated, there must have been similar words shared by other participants in the FGDs.

Discussion (page 21); the paragraph about U=U that describes reversal of “existential foreclosure, restoring marriage and parenthood aspirations” No participant in the results mentions parental aspirations, could you provide where this is coming from?

**Reviewer #2** : Please see the uploaded review for detailed comments. Overall, this is an important and timely manuscript that addresses mental health support for adolescents living with HIV in a resource-limited setting. The manuscript could be strengthened. I have no additional concerns about dual publication or publication ethics. The authors have provided a data availability statement, given the sensitivity of the qualitative data.

**Reviewer #3:**  Peer Review of Manuscript PMEN-D-26-00136

Thank you for inviting me to review this manuscript. The study addresses a highly important topic and has potential to make a meaningful contribution to the literature on adolescent HIV and mental health in Africa. However, several methodological, analytical, and interpretive issues need to be addressed before the manuscript can be considered for publication.

Major Comments

Title: From Suicidal Ideation to Hope: Mental Health Transformation Through a Culturally Adapted Multiple Family Group Therapy for Adolescents Living with HIV in Ghana

*The title does not fully adhere to epidemiological reporting principles. Scientific titles should clearly convey who was studied, what was investigated, where the study was conducted, and how the study was performed. While the current title identifies the population (adolescents living with HIV) and setting (Ghana), it does not indicate the study design. Readers cannot determine from the title whether this was a qualitative study, a process evaluation, or a randomized trial.

*The phrases "From Suicidal Ideation to Hope," "Mental Health Transformation," and particularly "Through a Culturally Adapted Multiple Family Group Therapy" imply a causal relationship between the intervention and participant outcomes. However, the qualitative design does not permit causal inference or demonstration of effectiveness of intervention.

Abstract

The abstract currently contains phrases such as: "complete mental health transformation"

"suicidal ideation cessation" These should be softened to avoid overstating findings.

Methods

*The manuscript is presented as a qualitative process evaluation nested within a randomized controlled trial. However, the parent trial itself is insufficiently described. Readers need a clearer understanding of the trial design, intervention and control conditions, participant flow, theoretical framework, primary outcomes, and current status of the trial. Without this context, it is difficult to interpret the significance of the qualitative findings or understand how the process evaluation contributes to the broader evaluation of the intervention.

*The intervention is described only briefly. Given that the central claim of the manuscript concerns “mental health transformation” following participation in the intervention, greater detail is needed regarding the structure and content of the Multiple Family Group Therapy (MFGT). The authors should use the TIDieR checklist to describe the intervention.

*A related concern is the absence of participants from the control arm. If this is a process evaluation embedded within a randomized trial, the rationale for interviewing only intervention participants should be explained.

Without interviewing control participants, they cannot determine whether the observed changes arose from: MFGT-specific techniques, common therapeutic factors (hope, attention, support, group belonging), natural adaptation to diagnosis, ongoing HIV care, or broader contextual influences.

*Adolescents in both intervention and control conditions may experience improvement over time, and many therapeutic mechanisms identified in this study (e.g., hope, group cohesion, universality, emotional expression, supportive relationships, and attention from facilitators) are common therapeutic factors that may operate across a range of psychosocial interventions. Without data from control participants, it is difficult to determine whether the reported changes are specific to MFGT or reflect non-specific therapeutic mechanisms that would have occurred in any supportive group context.

A process evaluation nested within a trial is most informative when it helps explain why the intervention worked better (or did not work better) than the comparison condition. At present, the manuscript largely documents that participants liked the intervention and perceived benefit, but it does not convincingly identify what was unique about MFGT or what its active ingredients were.

*Given that MFGT is fundamentally a family-based intervention, more information is needed regarding who participated in each family group. The manuscript should clarify the composition of groups, including which family members attended, how caregivers were involved, whether all sessions included both adolescents and caregivers, and how family interactions were incorporated into therapeutic activities. This information is critical for understanding what distinguishes the intervention from adolescent-only group approaches and for interpreting the reported improvements in family relationships and psychological wellbeing.

*The study is based on: Three focus group discussions,19 participants, Immediate post-intervention interviews, No comparison group, No quantitative mental health assessment.

*Therefore, the authors cannot conclude that: suicidal ideation was eliminated, anxiety was eliminated, suicide was prevented, all participants achieved transformation. The data support participants' perceptions of improvement, not objective evidence of clinical recovery.

*Sampling Strategy Requires Clarification. Participants were intervention completers only, attended at least 7 sessions, volunteered for FGDs. This likely excludes adolescents who dropped out, dissatisfied participants, participants who benefited least. As a result, findings may disproportionately reflect positive experiences. Authors should discuss how excluding non-completers may have biased findings.

Figures and Tables

Figure 2 ("psychological change mechanism") appears to imply an established causal pathway which cannot be supported by qualitative findings without a control group

Discussion

*The Discussion introduces concepts such as: "compounded developmental trauma" "existential foreclosure" "family betrayal”. These are powerful concepts but appear largely researcher-generated rather than participant-derived. The manuscript sometimes shifts from qualitative description into theoretical interpretation without sufficient evidence.

*For example: "stigma as familial betrayal" is a strong interpretation based on very limited participant quotations. Authors should distinguish participant experiences, researcher interpretations, and theoretical explanations. Avoid introducing new psychological constructs unless strongly supported by data.

*The manuscript states: "All adolescents who reported pre-intervention crying described complete cessation." "Complete resolution of suicidal ideation, anxiety, emotional dysregulation and HIV-related preoccupation across all participants." The qualitative findings do not provide sufficient evidence for such universal statements.

*No systematic assessment was conducted to verify: all participants improved, symptoms disappeared, effects were sustained.

The discussion would also benefit from a more detailed examination of the intervention techniques that may have contributed to perceived changes. For example, was change primarily driven by psychoeducation, peer support, family communication exercises, normalization of HIV, emotional expression, social learning, behavioral rehearsal, or other therapeutic processes? At present, the manuscript presents broad themes but does not adequately identify the active ingredients of the intervention.

*The manuscript repeatedly refers to "mechanisms of change," yet no formal mechanism evaluation was conducted. The qualitative data allows the authors to identify participant-perceived contributors to change, but they do not establish which intervention components were causally responsible for outcomes. The discussion should therefore be more cautious in distinguishing between perceived mechanisms and empirically demonstrated mechanisms

*The discussion should also be strengthened by comparing the findings with previous family-based and group-based HIV mental health interventions conducted in Africa, such as the VUKA Family Program in South Africa, CHAMP-related interventions, family strengthening interventions, and other group psychotherapy studies among adolescents living with HIV. Such comparisons would help situate the findings within the existing African evidence base and clarify the unique contribution of this intervention.

The Discussion compares findings primarily with: group therapy theory, family systems theory, Western literature. The paper would be strengthened by discussing: VUKA Family Program (South Africa), CHAMP interventions, Family Strengthening Intervention for HIV-affected families, Group Support Psychotherapy studies from Uganda, other African family-based HIV mental health interventions.

The manuscript states that interviews occurred immediately after intervention completion. Immediate post-intervention interviews often capture enthusiasm, emotional arousal, recency effects. They do not establish durability of change. The authors should discuss this as a limitation

The authors state: "Thematic saturation was achieved with 19 participants." Given the breadth of themes discussed: suicidality, family relationships, stigma, hope, adherence, mental health, the assertion of saturation requires stronger justification. Describe: how saturation was monitored, which coding framework was used, how the team determined no new information was emerging.

Minor Comments

1. Trial registration occurred in November 2024. Clarify whether registration was prospective relative to participant enrollment.

2. Participants include individuals up to age 22. Explain why they are classified as adolescents.

**********

what does this mean?). If published, this will include your full peer review and any attached files.

**Do you want your identity to be public for this peer review?**  If you choose “no”, your identity will remain anonymous but your review may still be made public.

For information about this choice, including consent withdrawal, please see our Privacy Policy

Reviewer #1: No

Reviewer #2: No

Reviewer #3: **Yes:**  Etheldreda Nakimuli Mpungu

**********

[NOTE: If reviewer comments were submitted as an attachment file, they will be attached to this email and accessible via the submission site. Please log into your account, locate the manuscript record, and check for the action link "View Attachments". If this link does not appear, there are no attachment files.]

**Figure Resubmissions:**

# Author comment: Author response to Decision Letter 1

**DOI:** 10.1371/journal.pmen.0000600.r002

15 Aug 2026

Attachment Submitted filename: Response_to_Reviewers_Plos Mental Health.docx

# Peer review: Decision Letter 1

**DOI:** 10.1371/journal.pmen.0000600.r003

**Authors:** Karli Montague-Cardoso

29 Sep 2026

PMEN-D-26-00136R1

From Suicidal Ideation to Hope: Mental Health Transformation Through a Culturally Adapted Multiple Family Group Therapy for Adolescents Living with HIV in Ghana

PLOS Mental Health

Dear Dr. Adjorlolo,

Thank you for submitting your revised manuscript to PLOS Mental Health. The Editors and Reviewers found the manuscript to be improved considerably and there are only two minor issues that we would like you to please revise prior to acceptance of the article. You can find those comments below. Thank you for your understanding.

Please submit your revised manuscript by Oct 28 2026 11:59PM. If you will need more time than this to complete your revisions, please reply to this message or contact the journal office at mentalhealth@plos.org. When you're ready to submit your revision, log on to https://www.editorialmanager.com/pmen/ and select the 'Submissions Needing Revision' folder to locate your manuscript file.

Please include the following items when submitting your revised manuscript:

- A letter that responds to each point raised by the editor and reviewer(s). You should upload this letter as a separate file labeled 'Response to Reviewers'.
- A marked-up copy of your manuscript that highlights changes made to the original version. You should upload this as a separate file labeled 'Revised Manuscript with Track Changes'.
- An unmarked version of your revised paper without tracked changes. You should upload this as a separate file labeled 'Manuscript'.

Guidelines for resubmitting your figure files are available below the reviewer comments at the end of this letter.

As the corresponding author, your ORCID iD is verified in the submission system and will appear in the published article. PLOS supports the use of ORCID, and we encourage all coauthors to register for an ORCID iD and use it as well. Please encourage your coauthors to verify their ORCID iD within the submission system before final acceptance, as unverified ORCID iDs will not appear in the published article. *Only*  the individual author can complete the verification step; PLOS staff *cannot*  verify ORCID iDs on behalf of authors.

We look forward to receiving your revised manuscript.

Kind regards,

Karli Montague-Cardoso

Staff Editor

PLOS Mental Health

**Journal Requirements:**

If the reviewer comments include a recommendation to cite specific previously published works, please review and evaluate these publications to determine whether they are relevant and should be cited. There is no requirement to cite these works unless the editor has indicated otherwise.

Please review your reference list to ensure that it is complete and correct. If you have cited papers that have been retracted, please include the rationale for doing so in the manuscript text, or remove these references and replace them with relevant current references. Any changes to the reference list should be mentioned in the rebuttal letter that accompanies your revised manuscript. If you need to cite a retracted article, indicate the article’s retracted status in the References list and also include a citation and full reference for the retraction notice.

[Note: HTML markup is below. Please do not edit.]

Reviewers' comments:

Reviewer's Responses to Questions

**Comments to the Author**

Reviewer #3: (No Response)

**********

publication criteria?

Reviewer #3: Yes

**********

3. Has the statistical analysis been performed appropriately and rigorously?

Reviewer #3: Yes

**********

4. Have the authors made all data underlying the findings in their manuscript fully available (please refer to the Data Availability Statement at the start of the manuscript PDF file)?

The PLOS Data policy

Reviewer #3: No

**********

5. Is the manuscript presented in an intelligible fashion and written in standard English?

Reviewer #3: Yes

**********

**Reviewer #3:**  The authors have largely addressed the comments raised in the earlier review. I have two remaining concerns.

First, Figures 1 and 2 are blurred in the material provided, and I cannot read their contents. Please ask the authors to supply clear, high-resolution versions of both figures.

Second, the data availability statement says that the data are in the manuscript or a supplementary file, but I cannot find a link to the underlying data. Please ask the authors to provide a repository link to the de-identified raw data and update the statement accordingly.

**********

what does this mean?). If published, this will include your full peer review and any attached files.

**Do you want your identity to be public for this peer review?**  If you choose “no”, your identity will remain anonymous but your review may still be made public.

For information about this choice, including consent withdrawal, please see our Privacy Policy

Reviewer #3: **Yes:**  Etheldreda Nakimuli-Mpungu

**********

[NOTE: If reviewer comments were submitted as an attachment file, they will be attached to this email and accessible via the submission site. Please log into your account, locate the manuscript record, and check for the action link "View Attachments". If this link does not appear, there are no attachment files.]

**Figure Resubmissions:**

# Author comment: Author response to Decision Letter 2

**DOI:** 10.1371/journal.pmen.0000600.r004

15 Oct 2026

Attachment Submitted filename: Response to the Reviewers comments_PLOS MH.docx

# Editor report: Decision Letter 2

**DOI:** 10.1371/journal.pmen.0000600.r005

**Authors:** Karli Montague-Cardoso

20 Oct 2026

From Suicidal Ideation to Hope: Mental Health Transformation Through a Culturally Adapted Multiple Family Group Therapy for Adolescents Living with HIV in Ghana

PMEN-D-26-00136R2

Dear Dr. Adjorlolo,

We are pleased to inform you that your manuscript 'From Suicidal Ideation to Hope: Mental Health Transformation Through a Culturally Adapted Multiple Family Group Therapy for Adolescents Living with HIV in Ghana' has been provisionally accepted for publication in PLOS Mental Health.

Before your manuscript can be formally accepted you will need to complete some formatting changes, which you will receive in a follow up email. A member of our team will be in touch with a set of requests.

Please note that your manuscript will not be scheduled for publication until you have made the required changes, so a swift response is appreciated.

IMPORTANT: The editorial review process is now complete. PLOS will only permit corrections to spelling, formatting or significant scientific errors from this point onwards. Requests for major changes, or any which affect the scientific understanding of your work, will cause delays to the publication date of your manuscript.

If your institution or institutions have a press office, please notify them about your upcoming paper to help maximize its impact. If they'll be preparing press materials, please inform our press team as soon as possible -- no later than 48 hours after receiving the formal acceptance. Your manuscript will remain under strict press embargo until 2 pm Eastern Time on the date of publication. For more information, please contact mentalhealth@plos.org.

Thank you again for supporting Open Access publishing; we are looking forward to publishing your work in PLOS Mental Health.

Best regards,

Karli Montague-Cardoso

Staff Editor

PLOS Mental Health

***********************************************************

Reviewer Comments (if any, and for reference):
